# Induction of Tolerogenic Dendritic Cells by a Noncoding Oligonucleotide

**DOI:** 10.1002/eji.70081

**Published:** 2025-10-26

**Authors:** Kahkashan Kamal, James Trumbo, Elina Richardsdotter‐Andersson, Marie Wahren‐Herlenius, Anna‐Lena Spetz

**Affiliations:** ^1^ Department of Molecular Biosciences The Wenner‐Gren Institute, Stockholm University Stockholm Sweden; ^2^ Department of Medicine Karolinska University Hospital, Karolinska Institutet Solna Sweden

**Keywords:** dendritic cell, noncoding oligonucleotides, PD‐L1, tolerance, T_regs_

## Abstract

Tolerogenic dendritic cells (tolDCs) that dampen T cell responses can be induced from blood monocytes in vitro using factors such as Vitamin D3 (VitD), dexamethasone, IL‐10, or rapamycin. However, challenges remain in obtaining robust and efficient generation of cell therapy‐based tolDCs without compromising their viability. We recently reported that CCR2‐dependent recruitment of monocytic cells, with the capacity to dampen T‐helper responses, occurs in mice treated with a single‐stranded oligonucleotide (ssON). Here, we investigated the effects of this immunomodulatory noncoding ssON on differentiating human monocytes towards DC in the presence of IL‐4 and GM‐CSF (moDC). The moDC differentiated in the presence of ssON upregulated CD1a but also increased their expression of PD‐L1. The differentiation of monocytes to moDC in the presence of ssON introduced transcriptomic changes, many of which overlapped with VitD‐moDC and resulted in moDCs with altered lipopolysaccharide (LPS)‐responsiveness. Moreover, ssON‐moDC exhibited a low capacity to stimulate alloreactive T cells in vitro and instead promoted the induction of CD4^+^FoxP3^+^CD25^+^ T cells. Experiments using chemical reagents support a role for PPAR‐γ in the generation of ssON‐moDC. Collectively, our data show that monocytes differentiated with IL‐4, GM‐CSF, and ssON generate cells with phenotypic and functional characteristics of tolDCs.

## Graphical abstract

In this article, the authors elucidated the immunoregulatory role of an oligonucleotide (ssON) that favors the induction of human tolerogenic dendritic cells (DC). The tolerogenic profile was evidenced by reduced responsiveness to lipopolysaccharides (LPS) (**A**). Importantly, the tolerogenic DCs had upregulated PD‐L1 molecules and functionally inhibited the proliferation of alloreactive T cells and induced FoxP3^+^ Tregs (**B**). This study envisions the development of ssON as therapeutic for rebalancing overactive T‐helper cell responses.

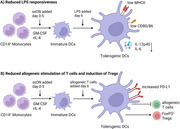

## 1 Introduction

Developing therapeutic approaches to achieve robust, long‐lasting, and antigen‐specific immune tolerance is a desirable goal to combat autoimmune or allergic immune reactions. DCs display phenotypic and functional heterogeneity and can be classified into plasmacytoid DCs, conventional types 1 and 2 DCs. In addition, moDCs adopt a DC‐like phenotype in inflammatory conditions. DCs reside in the periphery or blood in an immature state. DC activation by pathogens or danger signals results in the upregulation of molecules such as MHC, co‐stimulatory, adhesion, and proinflammatory cytokines [1]. However, certain stimuli, such as 1α,25‐dihydroxyvitamin D_3_ (VitD), Rapamycin, IL‐10, or corticosteroids, can transition DCs into a tolerogenic cell type, with the capability to induce anergy in T cells [2]. Such tolDCs exhibit an incomplete or altered maturation state upon stimulation. This is defined by low to intermediate levels of MHC‐class II expression and co‐stimulatory molecules, and high expression of checkpoint proteins [1]. In addition, they produce low levels of proinflammatory cytokines. TolDC‐based immunotherapy is an alternative to the continuous use of immunosuppressive drugs [3]. However, several challenges remain, such as protocol optimization [4] and low cell recovery due to VitD‐mediated cell death [5, 6].

We previously reported that a noncoding ssON inhibits TLR3 activation in moDC by temporarily inhibiting the uptake of ligands destined for endosomes expressing TLR3 [7]. We also showed that ssON dampened dsRNA‐mediated inflammation in the skin of nonhuman primates [7]. Recently, we showed that mice treated with ssON subcutaneously displayed CCR2‐dependent recruitment of cutaneous CD11b^+^Ly6C^+^ monocytic cells with tolerogenic properties, including expression of programmed death‐ligand 1 (PD‐L1) and immunoglobulin‐like transcript 3 [8]. As a logical next step, we investigated whether ssON can be used as an immunomodulator to induce tolerogenic human moDCs in vitro. There is currently no consensus for “gold standard” validation of tolDCs. We therefore conducted the commonly used methods and included analyses of cell surface markers, mixed lymphocyte reactions (MLR), and transcriptomic analyses.

## 2 Results and Discussion

### 2.1 MoDCs Cultured in the Presence of ssON Display Tolerogenic Features

**FIGURE 1:**
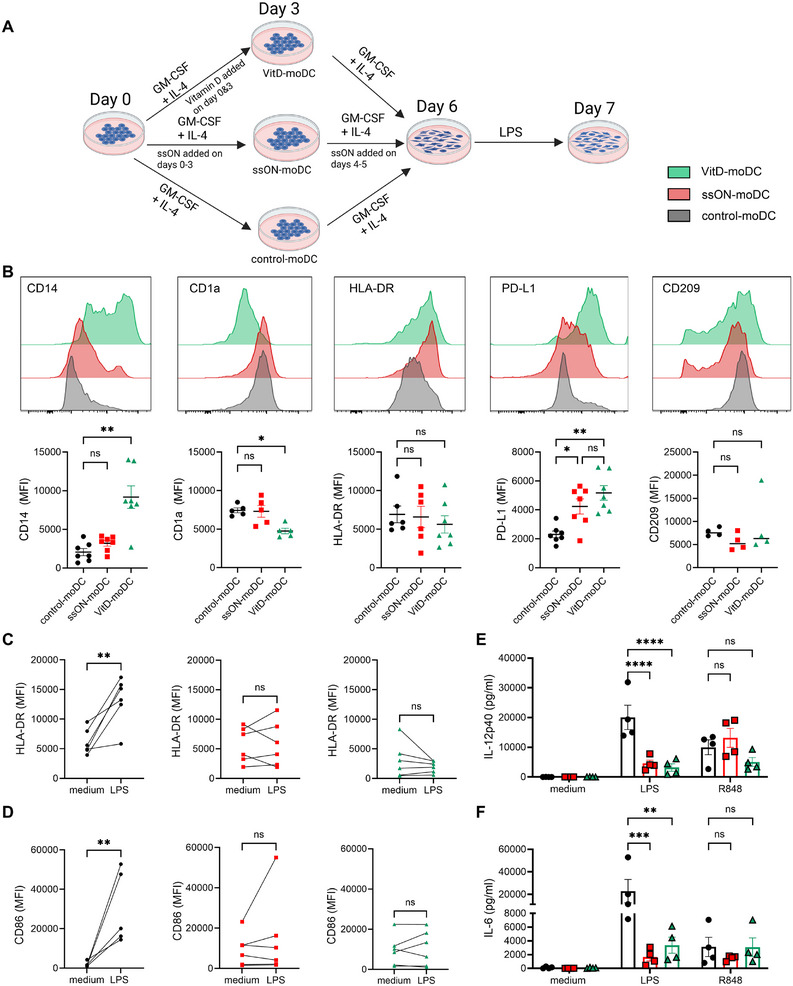
moDCs differentiated in the presence of ssON display tolerogenic features. (A) Schematic representation to generate moDCs. VitD was added on days 0 and 3 (10 nM), and ssON was added daily for 6 consecutive days (1 µM). (B) CD14, CD1a, HLA‐DR, PD‐L1, and CD209 expression in moDCs cultured in the presence or absence of VitD or ssON. (C) Expression of HLA‐DR and (D) CD86 in LPS‐stimulated mature moDCs. Quantities of (E) IL‐12p40 and (F) IL‐6 released in the supernatants after LPS‐ or R848‐stimulation. Data are representative of >3 independent experiments with *n *≥ 6 donors. Pairwise comparisons were made using the nonparametric Mann–Whitney test. Data are expressed as median (Histogram plots) or means ± SEM. Multiple comparisons were made using two‐way ANOVA (Tukey's test). ****p < 0.0001, *** *p < *0.001, **p < 0.01, and ns *p > *0.05.

Monocytes were cultured in the presence of GM‐CSF and IL‐4 to generate control‐moDCs. VitD is one way of inducing tolerogenic DCs; however, it causes considerable cell death (Figure S1) [9]. Nevertheless, we used VitD‐derived DCs as a comparator in assays with moDC differentiated in the presence of ssON (Figure 1A).

Phenotypic analyses of ssON‐moDCs revealed similar expressions of CD14, CD1a, and HLA‐DR molecules compared with control‐moDCs (Figure 1B). In contrast, VitD‐moDCs maintained high CD14 expression and did not upregulate CD1a [10]. However, we revealed increased PD‐L1 expression in both ssON‐ and VitD‐moDCs compared with control‐moDCs (Figure 1B).

The moDC generated in the presence of the classical GM‐CSF and rIL‐4 protocol are in an immature state with low expression of co‐stimulatory molecules. We therefore next stimulated with LPS, which resulted in the expected increase in expression of HLA‐DR and co‐stimulatory CD86, showing a transition toward a more mature phenotype (Figure 1C,D). We also investigated whether the LPS‐responsiveness changes when moDCs are differentiated in the presence of ssON or VitD. We report that upregulation of maturation markers was hampered in both ssON‐ and VitD‐moDCs (Figure 1C,D), indicating a tolerogenic profile. Control‐moDCs released substantial amounts of proinflammatory cytokines IL‐12p40 and IL‐6 upon LPS‐stimulation. In contrast, both ssON‐ and VitD‐moDCs produced significantly lower levels of these cytokines (Figure 1E,F). Albeit both ssON‐ and VitD‐moDCs were responsive to stimulation with the TLR7/8 agonist Resiquimod (R848) (Figure 1E,F). Slightly elevated quantities of IL‐10 were only observed in VitD‐moDCs, which were further increased upon LPS‐stimulation (Figure S2). Altogether, these data show that ssON‐moDCs display increased PD‐L1 expression and altered responsiveness to LPS‐stimulation, suggesting tolerogenic properties.

### 2.2 ssON‐moDCs Exhibit Reduced Capacity to Stimulate Alloreactivity and Instead Promote Tregs

**FIGURE 2:**
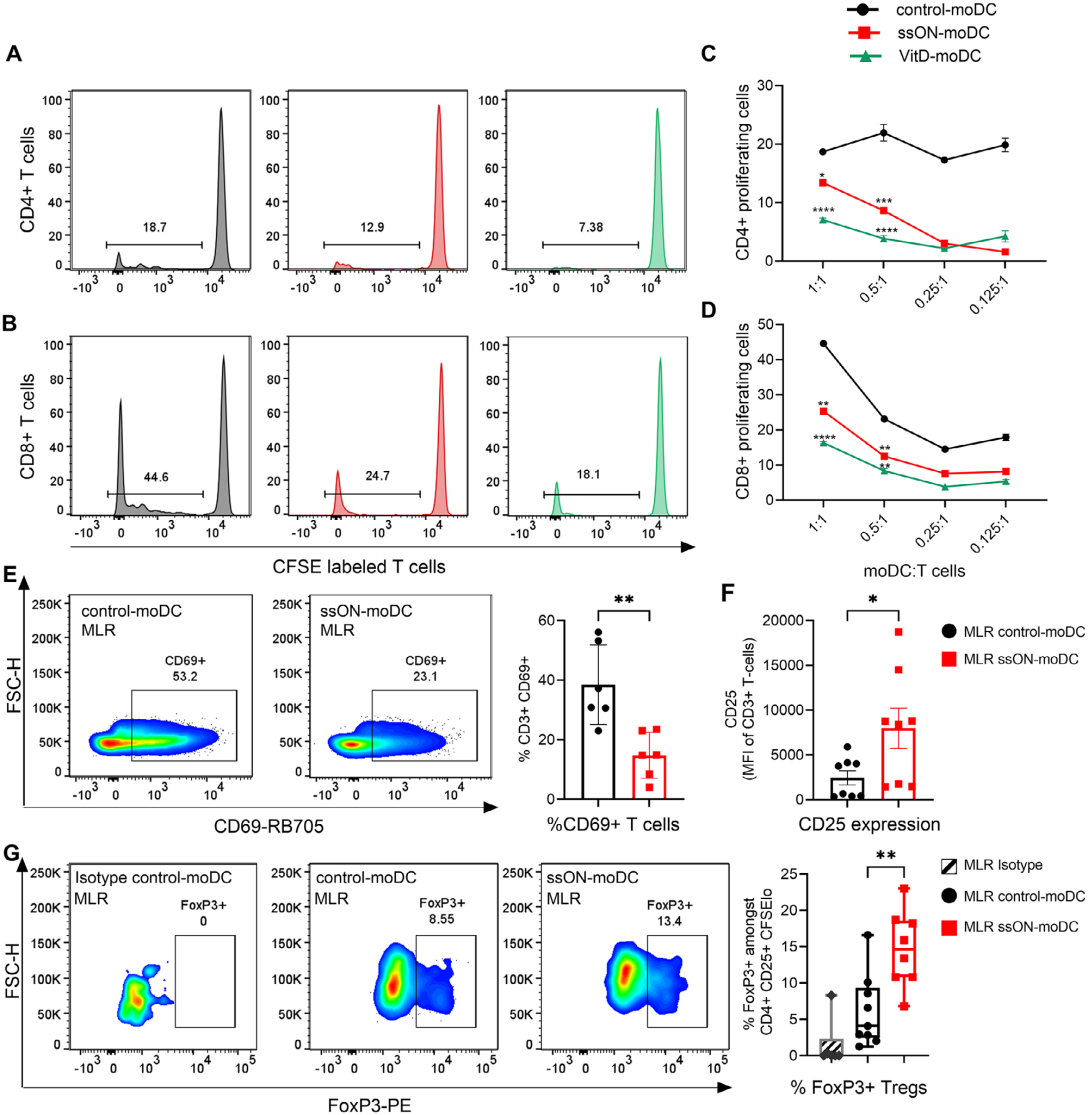
ssON‐moDCs provide poor stimulation of allogenic T cells and instead induce Tregs. CFSE‐dilution of allogeneic T cells co‐cultured with control‐moDCs (black), ssON‐moDCs (red), or VitD‐moDCs (green) for 6 days. Gating strategies are shown in Figures S3 and S4. The top and bottom panels show (A) CD4^+^ and (B) CD8^+^ T cells, respectively. The frequency of CFSE^low^ proliferating (C) CD4^+^ T cells and (D) CD8^+^ T cells at different ratios of moDCs. (E) Frequency of CD3^+^CD69^+^T cells among proliferating cells after 6 days of culture with either control‐ or ssON‐moDCs. (F) Mean Fluorescence intensities of CD25 expression in T cells proliferating after culture with allogeneic ssON‐moDCs or control‐moDC (G) FoxP3^+^ expression in T cells after culture with allogeneic ssON‐moDCs or control‐moDC at a 1:2 ratio. An isotype control was used to determine the background signal. Data were obtained by pooling from three independent experiments, including *n* = 6 individuals. Multiple comparisons were made using two‐way ANOVA (Tukey's test). Pairwise comparisons were made using the nonparametric Mann–Whitney test. Data are expressed as mean ± SEM. ****p < 0.0001, ***p < 0.001, **p < 0.01, *p < 0.05, and ns *p > *0.05.

A key characteristic of tolDCs is their poor activity as stimulators of allogeneic T cells. We found that both ssON‐ and VitD‐moDCs induced significantly lower proliferation of alloreactive CD4^+^ and CD8^+^ T cells than control‐moDCs (Figure 2A–D). The frequency of CD3^+^ T cells with upregulated expression of the early activation marker CD69 was higher when cultured with allogeneic control‐moDC compared with ssON‐moDC (Figure 2E and gating strategy in Figure S3A–C). We stimulated T cells with anti‐CD3 as a positive control for CD69 expression and used unstimulated T cells as a baseline control (Figure S3D,E). Conversely, we detected a significant increase in CD25 expression on CD3^+^ T cells (Figure 2F) and a higher frequency of FoxP3^+^ cells among CD4^+^CD25^+^CFSE^low^ cells in T cells cultured with allogeneic ssON‐moDC compared with allogeneic control‐moDC (Figure 2G; Figure S4A–C). We also assessed the expression of the T cell regulatory markers CTLA4 and LAG3 and did not detect any changes in their expression comparing T cells cultured with ssON‐moDCs or control‐moDCs (Figure S5A,B). Altogether, these data suggest that both ssON‐moDCs and VitD‐moDCs have reduced capacity to induce allogeneic T cell proliferation, especially CD4^+^ T cells. Furthermore, our data show that ssON‐moDC promotes the induction of CD4^+^FoxP3^+^CD25^+^ T cells in allogeneic cultures.

### 2.3 Tolerogenic Features of ssON‐moDCs Are Dependent on PPAR‐γ Activation

**FIGURE 3:**
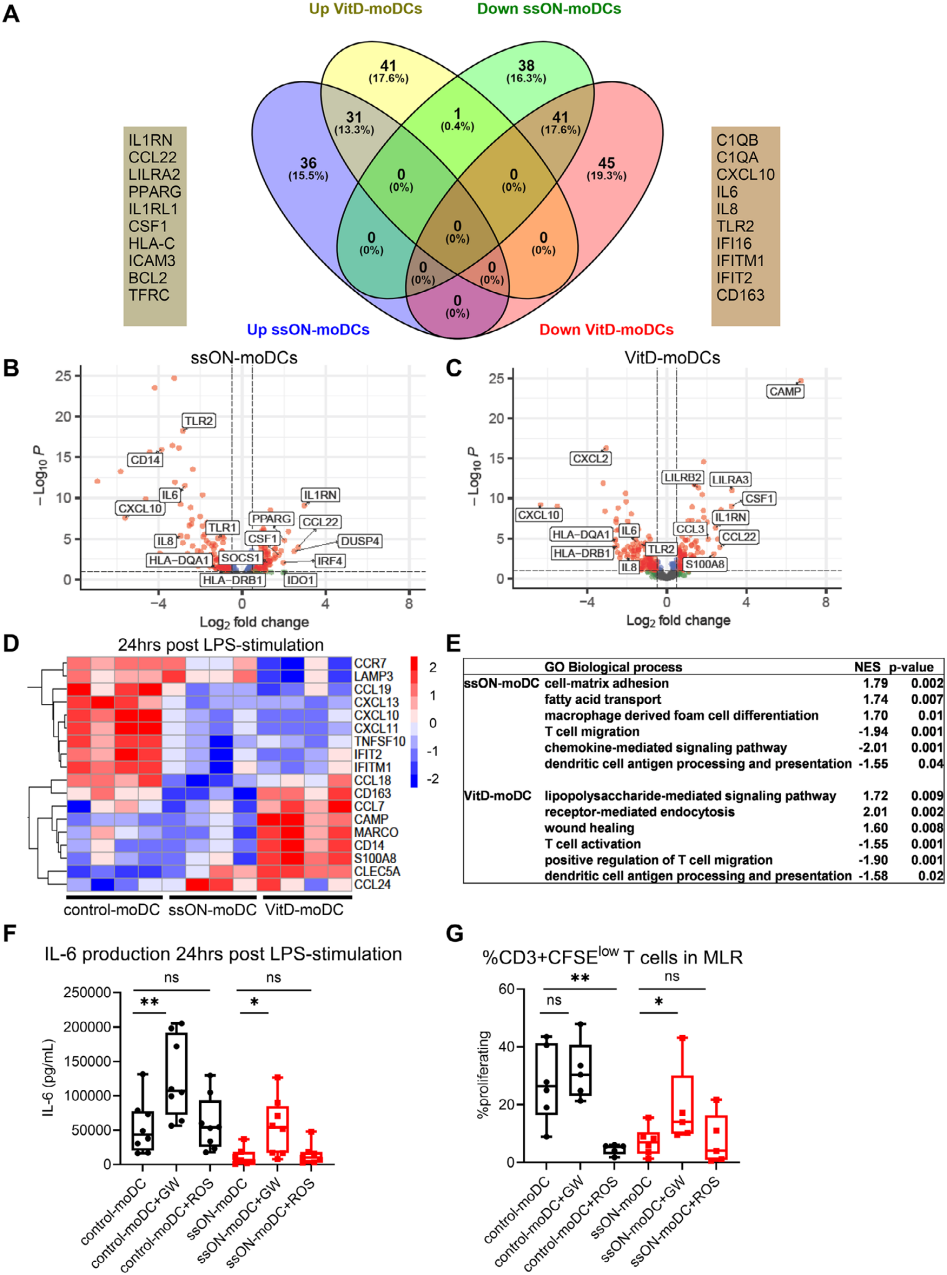
Transcriptomic profiling and modulation of PPAR‐γ reveal that tolerogenic features of ssON‐moDCs are dependent on PPAR‐γ activation. (A) A Venn diagram depicting the genes upregulated in both ssON‐ and VitD‐moDCs with Log2Foldchange>1 and adjusted *p‐*value < 0.05. The box on the left and right shows the topmost overlapping upregulated genes and downregulated genes, respectively. Volcano plots show the DEGs where the vertical and horizontal lines represent an absolute threshold cutoff of Log2Foldchange = 0.5 and adjusted *p‐*value = 0.01, respectively, in (B) ssON‐moDCs compared with control‐moDCs and (C) VitD‐moDCs compared with control‐moDCs. (D) Heatmap‐plot representing gene expression of topmost variable DEGs that were significantly changed (*p‐*value < 0.05) in LPS‐stimulated ssON‐ and VitD‐moDCs (when compared with LPS‐stimulated control‐moDCs). (E) Table showing gene ontology (GO) pathways (biological processes) associated with DEGs in LPS‐stimulated ssON‐ and VitD‐moDCs (when compared with LPS‐stimulated control moDCs) obtained using web‐based software NASQAR [18], including normalized enrichment score (NES) and *p‐*values associated with those pathways. (F) IL‐6 levels were measured in cell‐free supernatants after 24 h of LPS‐stimulation on day 7 in Control‐ and ssON‐moDCs that were differentiated in the presence of GW or ROS. (G) Frequencies of CFSE^low^ proliferating T cells after 6 days of co‐culture with different moDCs. Data are representative of *n* = 4–6 individuals obtained from 2 to 3 independent experiments. Pairwise comparisons were made using the nonparametric Mann–Whitney test. Multiple comparisons were made using one‐way ANOVA (Kruskal–Wallis test). Data are expressed as mean ± SEM. ** *p<*0.01, *p<0.05, and ns *p>*0.05.

To evaluate the transcriptomic programs associated with ssON‐ and VitD‐moDCs, we further assessed the differentially expressed genes (DEGs) relative to control‐moDCs. Based on the threshold criteria of Log2foldchange>1 and adjusted *p‐value*<0.05, out of 594 genes, we identified 101 and 110 DEGs in ssON‐ and VitD‐moDCs, respectively (entire lists in Tables S1 and S2). A Venn diagram shows 31 overlapping genes upregulated in both ssON‐ and VitD‐moDCs (Figure 3A), and among the top overlapping upregulated genes were *IL1RN*, *CCL22*, *LILRA2* (*ILT1*), and *PPARG* (Figure 3A; Table S3). IL1RN is an anti‐inflammatory cytokine, which inhibits the activity of IL‐1α and IL‐1β [11]. Both ssON‐ and VitD‐moDCs also produced increased levels of *CCL22*, a cytokine that promotes Treg communication [12]. The topmost‐downregulated overlapping genes included *C1QA/B, CXCL10, IL6, IL8, TLR2, CD163*, and several interferon‐stimulated genes (ISGs). Amongst the genes exclusively upregulated in ssON‐moDCs were *DUSP4*, a regulator of nucleic acid sensing [13], *SOCS1*, and *IRF4* (Figure 3B). Moreover, we detected a significant downregulation of PRRs, such as *CD14*, *TLR1*, and *TLR2* in ssON‐moDCs (Figure 3B,C).

We further investigated the transcriptomic changes associated with LPS‐stimulation of ssON‐ and VitD‐moDCs. A heatmap shows the topmost significantly variable genes (Figure 3D). *LAMP3*, a DC‐associated maturation marker, was downregulated efficiently in VitD‐moDCs, suggesting resistance to maturation. ISGs such as *IFIT2* and *IFITM1* were downregulated in LPS‐treated ssON‐ and VitD‐moDCs. In comparison to LPS‐stimulated control‐moDCs, LPS‐stimulated VitD‐moDCs show increased expression of scavenger receptors *CD163* and *MARCO*, which, on the contrary, were downregulated in ssON‐moDCs (Figure S6). Moreover, ssON‐moDCs maintained high expression levels of genes such as *PPARG*, *CD36*, and *CSF1* upon LPS stimulation (Figure S6). These genes showed a high enrichment score in GSEA analysis of upregulated pathways such as “fatty acid transport” and “macrophage‐derived foam cell differentiation” (Figure S3E; Table S4).

We also assessed the expression of additional tolerogenic markers LAP and CD276, as they were significantly upregulated in ssON‐moDCs at the mRNA level. Using flow cytometry, we confirmed the expression of these markers at the protein level (Figure S7). VitD‐moDCs displayed upregulation of pathways such as “Receptor‐mediated endocytosis”, “lipopolysaccharide‐mediated signaling pathway”, and “wound healing” after LPS‐stimulation (Figure 3E; Table S5). Pathways such as “T cell migration” and “T cell activation” were significantly downregulated in ssON‐moDCs and VitD‐moDCs, respectively. Pathways related to “dendritic cell antigen processing and presentation” were significantly downregulated in both ssON‐ and VitD‐moDCs. Altogether, the transcriptomic analyses showed that overlapping but also unique changes occurred in ssON‐ and VitD‐moDCs, which concurs with heterogeneous transcriptomic profiles of tolDCs generated by different pharmacological compounds that nevertheless share immunoregulatory properties.

To gain further mechanistic insights into the ssON‐mediated induction of a tolerogenic profile in moDC, we next performed experiments using either the activator (rosiglitazone (ROS)) or inhibitor (GW9662 (GW)) of PPAR‐γ [14]. The lipid‐activated transcription factor PPAR‐γ regulates lipid metabolism [15] and thereby indirectly modifies the immune phenotype in human DC development and maturation [16]. A growing body of evidence suggests that activation of PPARs (NF‐κB) pathways, consistent with reduced expression of proinflammatory cytokines [17]. We indeed found that inhibition of PPAR‐γ activity using GW increased the levels of IL‐6 produced in ssON‐moDCs after LPS‐stimulation (Figure 3F). Notably, the proliferation of allogeneic CD4^+^ T cells was also significantly restored in ssON‐moDC using GW (Figure 3G). The PPAR‐agonist ROS, conversely, prevented control‐moDC from inducing allogeneic T‐cell proliferation, and the inhibited allogeneic T cell proliferation in cultures with ssON‐moDC was maintained in the presence of ROS (Figure 3G). Altogether, the data using chemical reagents to modulate PPAR‐γ responses support a role for this transcription factor in the generation of ssON‐moDC.

### 2.4 Data Limitations and Perspectives

We here provide evidence of the involvement of PPAR‐γ in the induction of ssON‐moDC with reduced capacity to produce IL‐6 upon LPS‐stimulation and reduced capacity to support proliferation of alloreactive T cells. However, the upstream molecular events leading to activation of PPAR‐γ in ssON‐moDC are still elusive. We previously reported that ssON inhibits clathrin‐mediated endosomal uptake of ligands but leaves other endocytic routes functional [7]. It can be hypothesized that the ssON‐mediated temporal inhibition of clathrin‐mediated endocytosis changes the metabolic state of the moDC, shifting differentiation toward a tolerogenic profile.

### 2.5 Concluding Remarks

In this study, we identified a noncoding ssDNA that can induce tolDCs, as shown by its low allo‐stimulatory capacity and reduced LPS responsiveness. LPS‐stimulated tolDCs generated using ssON express higher levels of *PPARG*, in agreement with reduced expression of several proinflammatory cytokines. Inhibition of PPAR‐γ activity abrogated the ssON‐mediated differentiation to tolDCs in ssON‐moDC. Taken together, these data show the induction of human tolDCs by ssON, supporting our previous data in macaques [7] and mice [8] that ssON holds the potential as a novel therapeutic strategy for inflammatory disorders.

## 3 Materials and Methods

### 3.1 Immunomodulators

A 35‐base‐long, fully phosphorothioate‐modified ssON, with the sequence: 5’‐GAAGTTTTGAGGTTTTGAAGTTGTTGGTGGTGGTG‐3’ was purchased from Integrated DNA Technologies. Crystalline 1,25(OH)_2_D3 (VitD) obtained from Sigma (D1530) was reconstituted in ethanol and stored at −20°C. VitD was freshly diluted before each experiment, and the ethanol concentration in the experimental conditions did not exceed 0.0001%.

### 3.2 Cell Cultures

Human monocytes were isolated from buffy coats using RosetteSep Monocyte Enrichment kit (StemCell) and differentiated into moDCs using GM‐CSF (250 ng/mL, Peprotech) and IL‐4 (6.5 ng/mL, R&D Systems) for 6 days as previously described [7]. 1 µM of ssON was added daily from days 0 to 6, and 10 nM of VitD was added on days 0 and 3 to the cultures. Viable cell counts were determined by using the trypan blue staining method. For PPARγ modulation experiments, 10 µM of PPARγ inhibitor GW9662 or 10 µM of PPARγ activator rosiglitazone was added to the cultures on day 0. The medium was replenished on day 3. On day 6, the moDCs were stimulated with 10 ng/mL of LPS (Sigma) or 1 µg/mL R848 (InvivoGen). After 24 h of stimulation, the cell‐free supernatants were collected for ELISA analysis, and cells were harvested for flow cytometric analysis.

### 3.3 Flow Cytometry

A list of monoclonal antibodies is shown in Table S6. Dead cells were excluded by using LIVE/DEAD Fixable IR or Aqua Cell Stain Kit (ThermoFisher). Intranuclear staining was performed using Invitrogen FoxP3/transcription factor staining kit (Cat#00‐5523‐00) following the manufacturer's protocol. The samples were acquired on a BD FACSverse machine, and all analysis was performed with FlowJo software (Tree Star).

### 3.4 ELISA

Cell‐free supernatants were analyzed for IL‐6, IL‐10, and IL‐12p40 content using ELISA kits using the manufacturer's protocol (Mabtech).

### 3.5 MLR

T cells were isolated from cryopreserved PBMCs using the EasySep Human T Cell Isolation Kit from StemCell Technologies and labeled with 1 µM CFSE (ThermoFisher) for 8 min at room temperature. 100,000 CFSE‐labeled allogeneic T cells were added to each well in a 96‐well round‐bottom plate containing moDCs in a 1:1, 0.5:1, 0.25:1, or 0.125:1 ratio of moDCs to T cells. The co‐cultures were incubated for 5–6 days at 37°C, 5% CO_2_.

### 3.6 NanoString

RNA was extracted from the cells using the RNeasy Plus Mini kit (Qiagen). 100 ng of RNA was mixed with the reagents for NanoString analysis using the Human nCounter Immunology Panel and run on the nCounter Prep Station. Quality control and data normalization (to housekeeping genes) were performed on the count data using the nSolver Analysis software 4.0 (NanoString Technologies). Differential gene expression analysis was done using the DESeq2 package in R as previously described [8]. Volcano plots were constructed using the EnhancedVolcano function in R, where a Log2FoldChange = 0.5 and *p‐*value = 0.01 were set. Heatmaps were generated by regularized log transformation of the data and using the TopVarGenes function to visualize the most variable genes. Gene Ontology analysis was done to visualize enriched terms using the web‐based gene set enrichment analysis software NASQAR [18].

### 3.7 Data Analysis and Statistics

All data were analyzed using the GraphPad Prism software version 10. Pairwise comparisons between groups were made using the nonparametric Mann–Whitney test. Comparisons between multiple groups were made using the nonparametric one‐way ANOVA Kruskal–Wallis test, or two‐way ANOVA (Tukey's test).

### 3.8 Ethics Statement

Buffy coats were obtained from Karolinska University Hospital. Buffy coats are not subject to informed consent.

## Author contributions

Kahkashan Kamal designed and performed the moDC experiments and analyzed the data. James Trumbo assisted in moDC experiments. Elina Richardsdotter‐Andersson performed the NanoString assay. NanoString data were analyzed and interpreted by Kahkashan Kamal. Anna‐Lena Spetz and Marie Wahren‐Herlenius supervised the study. Kahkashan Kamal and Anna‐Lena Spetz wrote the paper. All authors discussed the results and provided comments and feedback.

## Conflicts of Interest

A.L.S. declares ownership in TIRmed Pharma, having IPR related to ssON. The remaining authors declare no conflicts of interest.

## Peer Review

The peer review history for this article is available at https://publons.com/publon/10.1002/eji.70081.

## Supporting information




**Supporting File 1**: eji70081‐sup‐0001‐SuppMat.pdf.

## Data Availability

Data sharing is not applicable as no datasets were generated or analyzed during the current study.
