# Supplementary material for: Induction of Tolerogenic Dendritic Cells by a Noncoding Oligonucleotide
Source: Eur J Immunol. 2025 Oct 26;55(10):e70081. doi: 10.1002/eji.70081 (PMC12555023; doi:10.1002/eji.70081)
Supplement: Supplementary file 1 — Supporting File 1: eji70081‐sup‐0001‐SuppMat.pdf. [file EJI-55-e70081-s001.pdf]

Supporting information TABLE 1**.** Differentially expressed gene in ssON-moDCs (Log2foldchange>1 and adjusted *p-value*<0.05) compared to control-moDCs.

Gene	Log2FoldChange	*p-value*
IL1RN	2.98	8.27443256829544e-10
CCL22	2.69	0.00010
DUSP4	2.49	0.00034
LILRA2	2.22	1.27099512934568e-06
PPARG	2.03	1.5029366757976e-05
IRF4	1.97	0.00825
CXCR4	1.87	0.00029
IL1RL1	1.83	6.15897659768174e-08
CSF1	1.76	0.00090
HLA-C	1.67	2.74765341801699e-06
ICAM3	1.61	2.84723854196169e-05
BCL2	1.59	0.00016
MS4A1	1.58	0.00089
TFRC	1.55	0.00017
CD9	1.54	4.58429737689308e-06
IL18R1	1.53	8.0535634208824e-06
MME	1.52	0.00322
ENTPD1	1.51	1.08369522517402e-05
STAT6	1.36	3.02979198732811e-09
CEACAM8	1.34	0.00513
TRAF1	1.33	0.01442
LILRA3	1.33	0.00581
CD83	1.32	0.00249
CD24	1.27	0.01472
SLAMF7	1.25	0.00462
CD1D	1.24	0.00083
CSF2RB	1.22	0.00036
BCL6	1.22	2.50249121954542e-05
CDKN1A	1.22	0.00057
SOCS1	1.22	0.00768
EGR2	1.14	0.00286
ITGAL	1.13	2.45616486377234e-05
TNFRSF1B	1.13	0.00077
CD79A	1.12	0.00012
ALAS1	1.11	0.00036
CD79B	1.10	0.00188
CD36	1.09	0.00910
G6PD	1.07	0.00012
LGALS3	1.06	0.01310
ITGAX	1.05	0.00038
GUSB	1.04	0.00626

PTPN6	1.04	8.5264220044564e-07
CCRL2	1.01	5.90953552240226e-07
TNFAIP3	1.01	0.00787
KLRD1	-1.01	0.01541
GBP1	-1.01	0.00057
FCER1G	-1.05	5.20423412879598e-07
ICAM1	-1.09	0.01514
SERPING1	-1.12	0.00228
TLR1	-1.15	8.41262527740984e-06
CYBB	-1.21	0.00341
IL1R2	-1.22	0.00365
CD4	-1.28	0.00225
CD59	-1.29	6.59697208869752e-05
CD28	-1.35	0.00070
CLEC4A	-1.36	0.00016
BST1	-1.38	3.78492520686236e-05
TNFSF10	-1.44	0.00919
BATF	-1.45	7.23600383208445e-05
VCAM1	-1.46	0.00091
IFITM1	-1.57	0.00106
NOD2	-1.60	3.30395823943553e-05
LILRA6	-1.68	1.014987446051e-07
PLAU	-1.71	1.68938403984601e-05
IL10	-1.80	3.00730566151844e-06
C1QA	-1.82	0.00010
FN1	-1.86	5.50263615917155e-06
CFD	-1.90	2.7107692781162e-07
PDCD1LG2	-1.90	4.17111954788111e-11
DPP4	-1.98	0.00039
CD209	-2.01	1.8169963139363e-08
FCGR2B	-2.14	0.00029
TGFBI	-2.37	3.05439880805843e-14
IFIT2	-2.37	7.33034499612323e-05
CFH	-2.41	4.42546279782383e-08
C1S	-2.42	1.59031540039005e-07
CTSC	-2.46	1.48183177057332e-09
CCL5	-2.53	2.31672015324013e-07
CCL2	-2.62	0.00067
C1QB	-2.63	5.34775281167531e-06
IL6	-2.74	2.90639561180395e-12
TLR2	-2.84	5.99329270537743e-19
MARCO	-2.84	3.00043351838585e-05
PPBP	-2.94	5.7442711575416e-10
IL8	-2.95	4.31370535348306e-06

CXCL2	-3.03	7.43777069845003e-17
FCGR3A/B	-3.22	1.1537366660062e-12
FCGR2A/C	-3.25	2.02385415128447e-25
C2	-3.33	3.66189850501651e-17
FCGR2A	-3.43	8.94327868324322e-26
CD14	-3.86	1.13735146897663e-16
CCL7	-3.93	0.00062
CCL23	-3.93	5.93868394628007e-16
LILRB5	-4.19	3.09613881759215e-24
CMKLR1	-4.28	3.87398282963374e-26
CXCL1	-4.42	2.31321015045282e-16
CCL13	-4.62	1.26852186724039e-10
CD163	-5.43	3.04291952067828e-33
CXCL10	-5.61	2.74651205091726e-08
CCL8	-5.82	5.74723187375206e-14
CCL18	-6.94	8.84199942826783e-13

* Transcriptomic Nanostring analyses of mRNA purified from four donors in two independent experiments showing differentially expressed genes of ssON-moDCs compared to control-moDCs from the same donors.

Supporting information TABLE 2. Differentially expressed gene in VitD-moDCs (Log2foldchange>1 and adjusted *p-value*<0.05) compared to control-moDCs*.

Gene	Log2FoldChange	*p-value*
CAMP	6.73	2.08897069954925e-25
LILRA3	3.27	9.63964198844347e-12
CSF1	3.24	9.54571323056191e-10
LILRA2	2.72	2.73190134130083e-09
CCL22	2.66	0.00013
ITGA6	2.56	1.17264076264759e-05
IL1RN	2.45	4.66223442902669e-07
S100A8	2.36	0.00090
CCL3	2.07	1.23847086992475e-05
GUSB	2.03	8.84108148858201e-08
TNFRSF1B	1.87	2.3418654243673e-08
CD97	1.84	2.59873934039731e-15
ALAS1	1.83	3.21370543672529e-09
HLA-C	1.82	2.89686543781723e-07
PLAUR	1.79	7.10316492112837e-07
ITGAL	1.77	3.27365570760195e-11
CD82	1.77	0.00054
LILRB2	1.59	4.65944380847634e-12
ITGAX	1.58	9.6312993000924e-08
TFRC	1.56	0.00015
C3	1.55	0.00054
ICAM3	1.49	0.00012
ENTPD1	1.47	2.0062618077639e-05
ITGB2	1.47	2.15194226577782e-06
IGF2R	1.43	2.67704693128534e-07
CCRL2	1.43	2.0820699553856e-12
CD9	1.40	3.30114243884931e-05
PPARG	1.38	0.00339
HLA-A	1.37	1.63363475999019e-12
BCL2	1.34	0.00148
LILRB4	1.31	0.00198
GZMB	1.31	0.00492
LILRB3	1.27	1.12671864773246e-09
STAT6	1.25	5.47095585686821e-08
CEBPB	1.24	6.46311241674858e-05
CD276	1.24	7.50243396943915e-05
FN1	1.23	0.00266
CD48	1.20	0.00107
ITGAM	1.19	3.21807474638381e-06
SLAMF7	1.16	0.00850
TNFSF15	1.13	0.01327

CD36	1.07	0.01027
BCAP31	1.07	6.36824387147241e-08
HLA-B	1.04	1.49048387085007e-05
IL1RL1	1.01	0.00412
TNFSF13B	-1.01	0.01007
IL12RB1	-1.04	5.06239964190418e-05
NOS2	-1.06	0.00454
TNFSF12	-1.08	0.00027
CD209	-1.09	0.00219
HLA-DMA	-1.09	3.61611308130864e-05
MAP4K1	-1.11	8.23660708240966e-05
BCL3	-1.16	0.00820
C1QA	-1.20	0.01044
CD163	-1.24	0.00513
CCL19	-1.28	0.01440
EDNRB	-1.28	0.00853
IL1RAP	-1.29	9.07407086725721e-05
TLR2	-1.29	4.39624017189769e-05
CFD	-1.42	0.00012
C2	-1.42	0.00026
CMKLR1	-1.43	0.00026
HLA-DMB	-1.43	6.57821250368118e-07
CD28	-1.44	0.00036
HLA-DRB3	-1.47	0.00120
LAG3	-1.47	0.00438
IL6	-1.50	3.06021415865405e-05
CLEC4A	-1.57	1.44781149418308e-05
IL1R2	-1.62	0.00013
C1S	-1.62	0.00031
CCR10	-1.66	0.00097
TNF	-1.68	0.00209
IL1R1	-1.69	0.00043
CD74	-1.71	5.63204449021557e-05
CXCR1	-1.74	0.00217
TCF4	-1.76	0.00035
TGFBI	-1.79	8.71209331239884e-09
IFITM1	-1.83	0.00016
LILRB5	-1.92	2.22696916923295e-07
CD70	-1.96	0.00010
CCR6	-1.97	7.18964776637937e-08
MX1	-1.97	0.00100
CTSC	-1.98	1.17153650476085e-06
IL8	-2.00	0.00183
FCGR2A	-2.06	2.59275825555911e-10

FCGR2A/C	-2.08	2.32958117226974e-11
IFIT2	-2.12	0.00041
FCGR2B	-2.15	0.00027
VCAM1	-2.21	2.03054577186482e-06
PPBP	-2.30	1.27364552123875e-06
HLA-DQB1	-2.33	0.00096
CTLA4_all	-2.36	0.00267
IL7R	-2.42	7.765475994279e-07
HLA-DRA	-2.45	1.50325422042447e-09
CCL13	-2.49	0.00052
HLA-DRB1	-2.50	7.60576994811843e-05
HLA-DPB1	-2.52	9.9503292083465e-07
HLA-DPA1	-2.54	3.99225467260948e-08
CCL23	-2.56	9.34407107792572e-08
CIITA	-2.59	1.32999467297425e-05
HLA-DQA1	-2.60	1.93068267727648e-05
TNFRSF4	-2.62	0.00011
C1QB	-2.81	1.22088101638738e-06
CTLA4-TM	-2.84	0.00070
CCL24	-2.86	0.00207
CXCL2	-3.06	5.26978447345246e-17
CXCL1	-3.11	3.81548650424323e-09
CFH	-3.20	1.32428311204793e-12
CD1A	-5.51	9.65467452363702e-10
CXCL10	-6.35	6.13687687878883e-10

* Transcriptomic Nanostring analyses of mRNA purified from four donors in two independent experiments showing differentially expressed genes of VitD-moDCs compared to control-moDCs from the same donors.

Supporting information TABLE 3. Venn Diagram genes

Genes included exclusively in "Up VitD-moDC":	Common genes in "Up ssON-moDC" and "Up VitD-moDC":	Genes included exclusively in "Up ssON-moDC":	Genes included exclusively in "Down VitD-moDC":	Common genes in "Down ssON-moDC" and "Down VitD-moDC":	Genes included exclusively in "Down ssON-moDC":
CAMP	IL1RN	DUSP4	MIF	IFI16	LCP2
ITGA6	CCL22	IRF4	ARHGDIB	HLA-DMB	CCR5
S100A8	LILRA2	CXCR4	SKI	MRC1	STAT1
CCL3	PPARG	MS4A1	TGFBR2	BLNK	BST2
PLAUR	IL1RL1	IL18R1	CASP1	TLR5	KCNJ2
CD82	CSF1	MME	CISH	FCGRT	TAGAP
LILRB2	HLA-C	CEACAM8	NT5E	IFI35	DEFB103B
C3	ICAM3	TRAF1	FAS	DEFB1	CFI
ITGB2	BCL2	CD83	CD160	MAF	CLU
IGF2R	TFRC	CD24	CCL11	GBP1	PRF1
LILRB4	CD9	CD1D	MBL2	IL1R2	IFNGR1
GZMB	ENTPD1	CSF2RB	TNFSF13B	CD28	PTAFR
LILRB3	STAT6	BCL6	IL12RB1	CLEC4A	CLEC7A
CEBPB	LILRA3	CDKN1A	NOS2	VCAM1	HFE
CD48	SLAMF7	SOCS1	TNFSF12	IFITM1	KLRD1
ITGAM	ITGAL	EGR2	HLA-DMA	C1QA	FCER1G
TNFSF15	TNFRSF1B	CD79A	MAP4K1	CFD	ICAM1
ARG2	CD36	CD79B	BCL3	PDCD1LG2	SERPING1
IRAK1	ITGAX	LGALS3	CCL19	CD209	TLR1
LTB4R	PTPN6	TNFAIP3	EDNRB	FCGR2B	CYBB
ICAM2	CCRL2	IRF3	IL1RAP	TGFBI	CD4
LAIR1	ITGAE	CD244	HLA-DRB3	IFIT2	CD59
IKBKE	HLA-A	TNFSF11	LAG3	CFH	BST1
IRF8	CD97	IKZF2	CCR10	C1S	TNFSF10
ITGA5	MAP4K2	MAP4K4	TNF	CTSC	BATF
IL6R	CD276	APP	IL1R1	C1QB	NOD2
CTSS	BCAP31	TNFSF4	CD74	IL6	LILRA6
CD99	MBP	FYN	CXCR1	TLR2	PLAU
ATG7	TGFB1	NFIL3	TCF4	PPBP	IL10
TAP2	HLA-B	TYK2	CD70	IL8	DPP4
CUL9	TAPBP	IRF5	CCR6	CXCL2	CCL5
SRC		RELA	MX1	FCGR2A/C	CCL2
PTPN22		IKBKG	HLA-DQB1	C2	MARCO
GPI		IKZF1	CTLA4_all	FCGR2A	FCGR3A/B
IL4R		TOLLIP	IL7R	CCL23	CD14
IKBKAP		TRAF6	HLA-DRA	LILRB5	CCL7
MYD88			HLA-DRB1	CMKLR1	CCL8
PTK2			HLA-DPB1	CXCL1	CCL18

IFNAR2			HLA-DPA1	CCL13	
B2M			CIITA	CD163	
			HLA-DQA1	CXCL10	
			TNFRSF4		
			CTLA4-TM		
			CCL24		
			CD1A		

Supporting information TABLE 4. GO pathways in LPS-stimulated ssON-moDCs

Description	NES	*p-value*	core enrichment genes
cell-matrix adhesion	1.796962	0.002899	CSF1/CD36/ITGAX/ITGAE/ITGAM/THY1/ITGA6/BCL2/MAP4K4/PLAU/ITGA4/BCL6/ITGB2/ITGAL
fatty acid transport	1.744867	0.007026	CD36/PPARG/SYK/TNFRSF11A
macrophage derived foam cell differentiation	1.703567	0.01171	CSF1/CD36/PPARG
foam cell differentiation	1.703567	0.01171	CSF1/CD36/PPARG
integrin-mediated signaling pathway	1.698821	0.005025	ITGAX/ITGAE/ITGAM/THY1/ITGA6/SYK/ITGA4/ITGB2/ITGAL/SRC/ITGB1/CD40LG
reactive oxygen species metabolic process	1.659182	0.008021	CD36/G6PD/IL19/TGFBR2/ITGAM/SYK/BCL2/ITGB2/TNF/TGFB1/TLR4/PRKCD
hematopoietic progenitor cell differentiation	1.612164	0.021226	PTPN6/KIT/BCL2/CSF1R/TGFB1/PDCD2/NOTCH1/TP53/TNFRSF13B/ABL1
regulation of reactive oxygen species metabolic process	1.596912	0.015831	CD36/G6PD/TGFBR2/ITGAM/SYK/BCL2/ITGB2/TNF/TGFB1/TLR4/PRKCD
regulation of cell-matrix adhesion	1.592498	0.020408	CSF1/CD36/THY1/BCL2/MAP4K4/PLAU/BCL6
protein dephosphorylation	1.568188	0.02799	LGALS3/PTPN6/PTPN22/DUSP4/PPIA/BCL2/TNF/TGFB1/IKBKB/PRKCD
response to lipoprotein particle	1.553663	0.037471	CD36/CD9/PPARG/SYK/ITGB2
cellular response to lipoprotein particle stimulus	1.553663	0.037471	CD36/CD9/PPARG/SYK/ITGB2
positive regulation of cell-matrix adhesion	1.547555	0.035294	CSF1/CD36/THY1/MAP4K4
cell-substrate adhesion	1.547292	0.0125	CSF1/CD36/ITGAX/ITGAE/ITGAM/THY1/ITGA6/BCL2/MAP4K4/PLAU/ITGA4/BCL6/ITGB2/ITGAL
organic acid transport	1.546132	0.040816	CD36/PPARG/SYK/TNFRSF11A/TNF
fatty acid metabolic process	1.522768	0.049412	CD36/PPARG/C3
receptor internalization	1.518691	0.048544	CD36/CD9/TFRC/SYK/ITGB2
positive regulation of reactive oxygen species metabolic process	1.5147	0.048544	CD36/TGFBR2/ITGAM/SYK/ITGB2/TGFB1/TLR4/PRKCD
positive regulation of calcium ion transport	-1.50213	0.039809	CCL4/LILRA5/CXCL9/CCL5/CXCL12/CXCL11/CXCL10

cell-cell signaling	-1.5095	0.009975	CCL7/IL6/PTGS2/CCL16/CCL4/C1QA/IFNG/CCL13/CXCL9/CCL5/TLR2/CXCL12/TNFAIP6/CCL23/CCL8/TNFSF10/CCL18/CXCL11/CXCL13/CXCL10
cellular response to interferon-gamma	-1.52712	0.021038	TLR3/CCL16/CCL4/IFNG/CCL13/GBP1/GBP5/CCL5/TLR2/CCL23/CCL8/CCL19/CCL18
defense response to other organism	-1.53733	0.001091	MX1/CLEC6A/IRF1/IFI35/CD209/PML/ARG1/FCER1G/IFNB1/KLRD1/CCL7/CD4/IL6/DEFB103A/CFH/NCR1/NLRP3/C1S/TLR3/CCL16/IFIH1/GZMB/SLAMF1/CCL4/C1QA/GNLY/CFD/CLEC4E/IFNG/C1R/LILRA5/FCGR2B/MARCO/NOD2/CCL13/C1QB/IL10/GBP1/GBP5/CXCL9/CCL5/TLR2/CFB/C2/IFITM1/SERPING1/CCL23/LAG3/PRF1/CCL8/IL27/IFIT2/CCL19/CCL18/CXCL11/CXCL13/CXCL10
positive regulation of leukocyte migration	-1.54914	0.016901	CCL7/IL6/SLAMF1/CCL4/CMKLR1/CCL5/CXCL12/CCL8/CCL19/CXCL13/CXCL10
dendritic cell antigen processing and presentation	-1.55328	0.048527	NOD1/CCR7/HLA-DRB1/CLEC4A/FCGR2B/NOD2/CCL19
response to interferon-gamma	-1.55354	0.019746	TLR3/CCL16/CCL4/IFNG/CCL13/GBP1/GBP5/CCL5/TLR2/IFITM1/CCL23/CCL8/CCL19/CCL18
regulation of granulocyte chemotaxis	-1.55759	0.033058	IL4/BST1/SLAMF1/DPP4/NOD2/CMKLR1/CCL5/TNFAIP6/CCL19
regulation of release of sequestered calcium ion into cytosol	-1.55931	0.039792	CXCL9/CXCL11/CXCL10
killing of cells of another organism	-1.56019	0.037479	DEFB103B/DEFB103A/GNLY/IFNG/CCL13/PRF1
negative regulation of interleukin-12 production	-1.56434	0.037479	JAK3/CCR7/SLAMF1/LILRA5/NOD2/IL10/CMKLR1
calcium ion transmembrane import into cytosol	-1.56977	0.025974	CXCL9/CCL19/CXCL11/CXCL10
positive regulation of ion transport	-1.57233	0.013393	CCL4/IFNG/LILRA5/KCNJ2/CXCL9/CCL5/CXCL12/CXCL11/CXCL10
leukocyte migration	-1.57341	0.002472	CCL16/VCAM1/SLAMF1/CCL4/DPP4/NOD2/CCL13/IL10/CXCL9/CMKLR1/CCL5/CXCL12/TNFAIP6/CCL23/CCL8/CCL19/CCL18/CXCL11/CXCL13/CXCL10
chronic inflammatory response	-1.5737	0.032368	TNFAIP3/IDO1/IL4/VCAM1/IL10/CCL5/CXCL13

regulation of leukocyte migration	-1.58732	0.006766	SLAMF1/CCL4/DPP4/NOD2/CMKLR1/CCL5/CXCL12/TNFAIP6/CCL8/CCL19/CXCL13/CXCL10
positive regulation of ion transmembrane transport	-1.59365	0.021488	IFNG/KCNJ2/CXCL9/CXCL11/CXCL10
chemotaxis	-1.60032	0.003755	IL4/DEFB103B/BST1/CCR8/SMAD3/FCER1G/CCL7/IL6/DEFB103A/CCL16/VCAM1/SLAMF1/CCL4/DPP4/NOD2/CCL13/IL10/CXCL9/CMKLR1/CCL5/CXCL12/TNFAIP6/CCL23/CCL8/CCL19/CCL18/CXCL11/CXCL13/CXCL10
positive regulation of cation transmembrane transport	-1.60266	0.021488	IFNG/KCNJ2/CXCL9/CXCL11/CXCL10
positive regulation of chemotaxis	-1.64407	0.004464	CCL7/IL6/SLAMF1/CCL4/CMKLR1/CCL5/CXCL12/CCL19/CXCL13/CXCL10
regulation of neutrophil chemotaxis	-1.64703	0.017301	XCL1/CCR7/BST1/DPP4/NOD2/TNFAIP6/CCL19
cellular response to virus	-1.64994	0.010769	IFNA2/TLR7/SMAD3/IFNB1/IL6/NLRP3/TLR3/IFIH1/IFNG/CCL5/CCL19/CXCL10
regulation of neutrophil migration	-1.66416	0.013115	XCL1/CCR7/BST1/DPP4/NOD2/TNFAIP6/CCL19
chemical homeostasis	-1.66748	0.005405	CCL7/IL6/CDH5/IFNG/KCNJ2/CCL13/CXCL9/CCL5/CXCL12/CCL23/CCL8/CCL19/CXCL11/CXCL10
humoral immune response	-1.69491	0.002551	IL6/CFH/C1S/C1QA/GNLY/CFD/IFNG/C1R/FCGR2B/NOD2/CCL13/C1QB/CXCL9/CFB/C2/SERPING1/CXCL11/CXCL13/CXCL10
positive regulation of lymphocyte migration	-1.70793	0.006557	CCL7/CCL4/CCL5/CXCL12/CXCL13/CXCL10
monocyte chemotaxis	-1.71505	0.003077	CCL7/IL6/CCL16/CCL4/CCL13/CCL5/CXCL12/CCL23/CCL8/CCL19/CCL18/CXCL10
mononuclear cell migration	-1.71812	0.002674	CCL16/SLAMF1/CCL4/CCL13/CMKLR1/CCL5/CXCL12/CCL23/CCL8/CCL19/CCL18/CXCL11/CXCL13/CXCL10
G protein-coupled receptor signaling pathway	-1.71899	0.00406	CCL16/CCL4/MARCO/CCL13/CXCL9/CMKLR1/CCL5/CXCL12/CCL23/CCL8/CCL19/CCL18/CXCL11/CXCL10
regulation of lymphocyte migration	-1.75166	0.003306	CCL7/CCL4/CCL5/CXCL12/CXCL13/CXCL10
cell chemotaxis	-1.76002	0.001271	CCL7/IL6/DEFB103A/CCL16/VCAM1/SLAMF1/CCL4/DPP4/NOD2/CCL13/IL10/CXCL9/CMKLR1/CCL5/CXCL12/TNFAIP6/CCL23/CCL8/CCL19/CCL18/CXCL11/CXCL13/CXCL10

positive regulation of T cell migration	-1.76528	0.003407	CCL5/CXCL12/CXCL13/CXCL10
cellular homeostasis	-1.76623	0.004231	MCL1/PML/CCL7/IL6/CDH5/IFNG/KCNJ2/CCL13/CXCL9/CCL5/CXCL12/CCL23/CCL8/CCL19/CXCL11/CXCL10
regulation of chemotaxis	-1.78572	0.002813	IL4/BST1/SMAD3/CCL7/IL6/SLAMF1/CCL4/DPP4/NOD2/CMKLR1/CCL5/CXCL12/TNFAIP6/CCL19/CXCL13/CXCL10
myeloid leukocyte migration	-1.79757	0.001289	CCL16/SLAMF1/CCL4/DPP4/NOD2/CCL13/CXCL9/CMKLR1/CCL5/CXCL12/TNFAIP6/CCL23/CCL8/CCL19/CCL18/CXCL11/CXCL13/CXCL10
regulation of lymphocyte chemotaxis	-1.81087	0.001733	CCL7/CCL4/CCL5/CXCL13/CXCL10
cellular chemical homeostasis	-1.82495	0.001406	IFNG/KCNJ2/CCL13/CXCL9/CCL5/CXCL12/CCL23/CCL8/CCL19/CXCL11/CXCL10
leukocyte chemotaxis	-1.83071	0.001292	CCL16/SLAMF1/CCL4/DPP4/NOD2/CCL13/IL10/CXCL9/CMKLR1/CCL5/CXCL12/TNFAIP6/CCL23/CCL8/CCL19/CCL18/CXCL11/CXCL13/CXCL10
regulation of T cell migration	-1.84128	0.001642	CCL5/CXCL12/CXCL13/CXCL10
positive regulation of leukocyte chemotaxis	-1.84474	0.00158	CCL7/IL6/SLAMF1/CCL4/CMKLR1/CCL5/CXCL12/CCL19/CXCL13/CXCL10
granulocyte chemotaxis	-1.87243	0.001412	CCL16/SLAMF1/CCL4/DPP4/NOD2/CCL13/CXCL9/CMKLR1/CCL5/TNFAIP6/CCL23/CCL8/CCL19/CCL18/CXCL11/CXCL13/CXCL10
granulocyte migration	-1.88722	0.001351	CCL16/SLAMF1/CCL4/DPP4/NOD2/CCL13/CXCL9/CMKLR1/CCL5/TNFAIP6/CCL23/CCL8/CCL19/CCL18/CXCL11/CXCL13/CXCL10
neutrophil migration	-1.90918	0.00141	CCL16/CCL4/DPP4/NOD2/CCL13/CXCL9/CCL5/TNFAIP6/CCL23/CCL8/CCL19/CCL18/CXCL11/CXCL13/CXCL10
neutrophil chemotaxis	-1.92702	0.001403	CCL16/CCL4/DPP4/NOD2/CCL13/CXCL9/CCL5/TNFAIP6/CCL23/CCL8/CCL19/CCL18/CXCL11/CXCL13/CXCL10
lymphocyte chemotaxis	-1.93298	0.001592	CCL16/CCL4/CCL13/CCL5/CCL23/CCL8/CCL19/CCL18/CXCL11/CXCL13/CXCL10
T cell migration	-1.94619	0.001592	CCL5/CXCL12/CXCL11/CXCL13/CXCL10
lymphocyte migration	-1.94756	0.001458	CCL16/CCL4/CCL13/CCL5/CXCL12/CCL23/CCL8/CCL19/CCL18/CXCL11/CXCL13/CXCL10

regulation of leukocyte chemotaxis	-1.98176	0.001488	CCL7/IL6/SLAMF1/CCL4/DPP4/NOD2/CMKLR1/CCL5/CXCL12/TNFAIP6/CCL19/CXCL13/CXCL10
antimicrobial humoral immune response mediated by antimicrobial peptide	-1.9891	0.001637	GNLY/NOD2/CCL13/CXCL9/CXCL11/CXCL13/CXCL10
chemokine-mediated signaling pathway	-2.01857	0.001473	CCL16/CCL4/CCL13/CXCL9/CMKLR1/CCL5/CXCL12/CCL23/CCL8/CCL19/CCL18/CXCL11/CXCL13/CXCL10
response to chemokine	-2.01857	0.001473	CCL16/CCL4/CCL13/CXCL9/CMKLR1/CCL5/CXCL12/CCL23/CCL8/CCL19/CCL18/CXCL11/CXCL13/CXCL10
cellular response to chemokine	-2.01857	0.001473	CCL16/CCL4/CCL13/CXCL9/CMKLR1/CCL5/CXCL12/CCL23/CCL8/CCL19/CCL18/CXCL11/CXCL13/CXCL10
antimicrobial humoral response	-2.02519	0.001605	GNLY/NOD2/CCL13/CXCL9/CXCL11/CXCL13/CXCL10

Supporting information TABLE 5. GO pathways in LPS-stimulated VitD-moDCs

Description	NES	*p-value*	core enrichment genes
membrane organization	2.135038	0.002336	MARCO/C3/CD36/S100A9/ITGB2/ITGAM/CD9/CR1/MBP
integrin-mediated signaling pathway	2.083542	0.002273	FN1/ITGB2/ITGAM/ITGAX/ITGA6/ITGAL/ITGAE/SYK/PTK2/ITGB1
regulation of cell shape	2.042596	0.002217	CSF1R/CCL24/FN1/CCL7/ITGB2/CCL2/CCL3/PTK2
endocytosis	2.014424	0.00271	CD14/MARCO/C3/CD36/MRC1/ITGB2/ITGAM/CD9/CLEC7A/HFE/IGF2R/LILRB1/TGFBR2/CD6/CD22/SYK/ITGB1/LGALS3/CD163/APP/TFRC/MSR1/CD209/CFP/PYCARD
phagocytosis	2.013632	0.002475	CD14/MARCO/C3/CD36/ITGB2/ITGAM/ITGAL/CLEC7A/CCL2/IL1B/PECAM1/ICAM3/SYK/PTK2/TLR4/ITGB1
receptor-mediated endocytosis	2.012374	0.002421	CD14/MARCO/C3/CD36/MRC1/ITGB2/ITGAM/CD9/HFE/IGF2R/LILRB1/TGFBR2
reactive oxygen species metabolic process	1.930612	0.002336	CD36/ITGB2/ITGAM/CLEC7A/G6PD/TGFBR2/TGFB1/CYBB/SYK/TLR4/MAPK14/BST1/IL19/ARG2
response to lipoprotein particle	1.919215	0.002179	CD36/ITGB2/CD9/PPARG/SYK/TLR4/ITGB1
cellular response to lipoprotein particle stimulus	1.919215	0.002179	CD36/ITGB2/CD9/PPARG/SYK/TLR4/ITGB1
superoxide metabolic process	1.882652	0.002179	CD36/ITGB2/ITGAM/CLEC7A/TGFB1/CYBB/SYK
regulation of reactive oxygen species metabolic process	1.859968	0.004684	CD36/ITGB2/ITGAM/CLEC7A/G6PD/TGFBR2/TGFB1/SYK/TLR4/MAPK14/BST1/ARG2
positive regulation of reactive oxygen species metabolic process	1.855683	0.004415	CD36/ITGB2/ITGAM/CLEC7A/TGFBR2/TGFB1/SYK/TLR4/MAPK14
vesicle-mediated transport	1.848188	0.002801	CD14/MARCO/C3/CD36/MRC1/ITGB2/ITGAM/ITGAL/CD9/CLEC7A/HFE/CCL2/IGF2R/LILRB1/TGFBR2/BCAP31/IL1B/CD6/MAP4K2/PECAM1/ICAM3/CD22/CCL3/SYK/PTK2/TLR4/ITGB1/LGALS3/CD163/APP/TFRC/MSR1/PTAFR/CD209/CFP/PYCARD

fatty acid transport	1.819749	0.002179	CD36/IL1A/IL1B/PPARG/SYK
acute-phase response	1.801394	0.004464	FN1/PTGS2/IL1A/IL1B/CD163/CEBPB/TFRC/IL6/SIGIRR/HAMP/TNF
receptor internalization	1.768112	0.004415	CD36/ITGB2/CD9/LILRB1/SYK/ITGB1/TFRC
nervous system development	1.761568	0.002841	C3/S100A8/CSF1R/CSF1/FN1/S100A9/ITGAM/ITGAX/ITGA6/CD9/CXCL1/CCL2/G6PD/TGFBR2/SPP1/EGR2/TGFB1/IL1B/MBP/TNFRSF1B/CCL3/IFNGR1/BCL6/PTK2/NCAM1/LIF/TLR4/ITGB1/PPIA/APP/SDHA/CEBPB
regulation of lipopolysaccharide-mediated signaling pathway	1.733724	0.008715	CD14/CD36/LILRA2
lipopolysaccharide-mediated signaling pathway	1.722862	0.009685	CD14/CD36/LILRA2/PTPN22/CCL2/TGFB1/IL1B/CD6/CCL3/TLR4/IRAK1/MAPK14/LY96/PTAFR
gliogenesis	1.719691	0.010127	S100A8/CSF1R/CSF1/S100A9/CD9/CCL2/EGR2/TGFB1/IL1B/TNFRSF1B/CCL3/IFNGR1/LIF/TLR4
neutrophil activation	1.717686	0.008929	CAMP/LILRA2/ITGB2/ITGAM
myeloid cell development	1.711184	0.011038	PTPN6/LILRB1/G6PD/BCL6/APP/CEBPB/ALAS1
cell-matrix adhesion	1.681412	0.007335	CD36/CSF1/FN1/ITGB2/ITGAM/ITGAX/ITGA6/ITGAL/PLAU/ITGAE/BCL6/PTK2/ITGB1
regulation of intracellular protein transport	1.671134	0.009112	CD36/ITGB2/ITGAM/PTGS2/TGFB1/BCAP31/IL1B
fatty acid metabolic process	1.670513	0.010799	C3/CD36/PTGS2/IL1B/PPARG/MAPK14
granulocyte activation	1.649134	0.009091	CAMP/LILRA2/ITGB2/ITGAM/CCL3/SYK
positive regulation of lipid localization	1.648146	0.015453	C3/CD36/SPP1/IL1A/IL1B/PPARG/MSR1/IKBKE
positive regulation of secretion	1.647138	0.018868	S100A8/ITGB2/ITGAM/HFE/SPP1/IL1A/TGFB1/IL1B/PPARG/SYK/TLR4/PPIA
positive regulation of protein localization	1.642782	0.014528	ITGB2/ITGAM/PTGS2/IL1A/TGFB1/BCAP31/IL1B/PECAM1/PPARG/LIF/TLR4/ITGB1/PPIA/LGALS3/MAPK14/TFRC

hemostasis	1.630908	0.016746	CD36/FN1/CD9/PTPN6/PLAU/PLAUR/GPI/SYK/TLR4/PPIA/ENTPD1/MAPK14
cell development	1.62839	0.002841	CLEC5A/C3/S100A8/CSF1R/FN1/S100A9/ITGA6/PTPN6/CLEC7A/LILRB1/G6PD/TGFBR2/SPP1/EGR2/IL1A/TGFB1/IL1B/MBP/TNFRSF1B/PECAM1/PPARG/IFNGR1/BCL6/PTK2/NCAM1/LIF/TLR4/ITGB1
regulation of intracellular transport	1.62821	0.013793	CD36/ITGB2/ITGAM/PTGS2/TGFB1/BCAP31/IL1B
positive regulation of growth	1.626934	0.013393	S100A8/CSF1/FN1/S100A9/TGFBR2
wound healing	1.616453	0.008197	S100A8/CD36/FN1/CD9/PTPN6/PLAU/PLAUR/CLEC7A/TGFBR2/IL1A/TGFB1/PPARG/SYK/PTK2/TLR4/ITGB1/PPIA/ENTPD1/MAPK14
platelet aggregation	1.609684	0.023965	FN1/CD9/PTPN6/SYK/PPIA
regulation of lipid localization	1.607621	0.013667	C3/CD36/SPP1/IL1A/IL1B/PPARG/SYK/MSR1/IKBKE/IL6
lipid localization	1.597281	0.023095	C3/CD36/SPP1/PTGS2/IL1A/IL1B/PPARG/SYK/MSR1/IKBKE/IL6
negative regulation of MAPK cascade	1.595262	0.028761	PTPN22/PTPN6/IRAK3/IL1B/PPARG/LIF/TLR4/PPIA
biological process involved in interaction with symbiont	1.593223	0.016092	CAMP/CSF1R/FN1/IGF2R/CARD9/CCL3
positive regulation of synaptic transmission	1.591644	0.030238	CCL2/PTGS2/LILRB2/APP/ABL1/CCR2/MAPK1/TNF/CX3CR1/MME
cell morphogenesis involved in neuron differentiation	1.582769	0.025	CSF1R/FN1/SPP1/EGR2/MBP/PTK2/NCAM1/ITGB1/APP
hematopoietic progenitor cell differentiation	1.582278	0.031042	CSF1R/PTPN6/TGFB1
regulation of secretion	1.578496	0.012376	S100A8/ITGB2/ITGAM/HFE/LILRB1/SPP1/IL1A/TGFB1/IL1B/TNFRSF1B/PPARG/SYK/LIF/TLR4/ADA/PPIA
lipid transport	1.578169	0.023419	CD36/SPP1/PTGS2/IL1A/IL1B/PPARG/SYK
pattern specification process	1.576286	0.020501	C3/ITGAM/TGFBR2/EGR2

organic acid transport	1.575103	0.035556	CD36/IL1A/IL1B/PPARG/SYK/ITGB1
blood coagulation	1.566929	0.025943	CD36/FN1/CD9/PTPN6/PLAU/PLAUR/SYK/TLR4/PPIA/ENTPD1/MAPK14
coagulation	1.566929	0.025943	CD36/FN1/CD9/PTPN6/PLAU/PLAUR/SYK/TLR4/PPIA/ENTPD1/MAPK14
interleukin-6 production	1.562068	0.007732	CD36/LILRA2/PTPN22/PTPN6/CLEC7A/POU2F2/IL1A/IL16/IRAK3/CARD9/LILRB2/IL1B/MBP/SYK/TLR4/TLR8/APP/CEBPB/TLR1/PTAFR/PYCARD/IL1RAP/BTK/IL6
regulation of interleukin-6 production	1.562068	0.007732	CD36/LILRA2/PTPN22/PTPN6/CLEC7A/POU2F2/IL1A/IL16/IRAK3/CARD9/LILRB2/IL1B/MBP/SYK/TLR4/TLR8/APP/CEBPB/TLR1/PTAFR/PYCARD/IL1RAP/BTK/IL6
positive regulation of gliogenesis	1.555142	0.035477	EGR2/TGFB1/IL1B/TNFRSF1B/LIF
platelet activation	1.550747	0.018391	FN1/CD9/PTPN6/SYK/TLR4/PPIA/MAPK14
T cell activation	-1.55781	0.001445	CD160/JAK3/KLRC1/DPP4/BCL3/RAG1/STAT3/JAK2/PTPN2/HLA-DQB1/IL7R/CD86/CTNNB1/XCL1/PTGER4/SLAMF7/SMAD3/TNFSF4/TRAF6/IL4/SLAMF1/ PDCD1LG2/IFNB1/CD274/IL15/IL7/CD74/HLA-DPB1/PRDM1/HLA-DQA1/HLA-DRA/HLA-DPA1/HLA-DRB1/HLA-DOB/CCR6/IL12B/RELB/IFNG/ARG1/VCAM1/SOCS1/TNFSF13B/TNFRSF9/IRF1/CCL5/TNFRSF4/CD83/GPR183/IL10/CASP3/CD70/CD80/IL2RA/IDO1/EBI3/IRF4/CCR7/IL27/LAG3/CCL19
calcium ion transport	-1.56992	0.016393	CCL5/CXCL12/CCR7/CXCL9/CCL19/CXCL11/CXCL10
regulation of neutrophil chemotaxis	-1.57534	0.039927	DPP4/XCL1/TNFAIP6/CD74/CCR7/CCL19
regulation of lymphocyte activation	-1.57877	0.00146	CD160/JAK3/KLRC1/DPP4/RAG1/JAK2/PTPN2/HLA-DQB1/IL7R/TNFAIP3/CD86/CTNNB1/XCL1/IL5/TNFSF4/CD40/TRAF6/IL4/SLAMF1/PDCD1LG2/IFNB1/CD274/IL15/IL7/CD74/HLA-DPB1/PRDM1/HLA-DQA1/HLA-DRA/HLA-DPA1/HLA-DRB1/HLA-DOB/IL12B/CDKN1A/IFNG/ARG1/VCAM1/SOCS1/TNFSF13B/TNFRSF9/IRF1/CCL5/TNFRSF4/CD83/GPR183/IL10/CASP3/CD70/CD80/IL2RA/IDO1/EBI3/IRF4/CCR7/IL27/LAG3/CCL19

antigen processing and presentation	-1.5792	0.021138	HLA-DQB1/NOD1/TRAF6/TAP1/CD74/HLA-DPB1/HLA-DQA1/HLA-DRA/HLA-DPA1/HLA-DRB1/HLA-DOB/RELB/CD1A/CCR7/CCL19
T cell differentiation	-1.58165	0.004615	JAK3/KLRC1/BCL3/RAG1/STAT3/PTPN2/IL7R/CD86/CTNNB1/PTGER4/TNFSF4/IL4/IFNB1/IL15/IL7/CD74/PRDM1/ HLADRA/HLADRB1/CCR6/IL12B/RELB/IFNG/SOCS1/TNFRSF9/IRF1/CD83/GPR183/CD80/IL2RA/IRF4/CCR7/IL27/LAG3/CCL19
humoral immune response	-1.58337	0.001575	C7/IFNB1/IL7/C9/CFD/C2/HLA-DRB1/CCR6/CFB/IFNG/CFH/CD83/GPR183/C1QB/SERPING1/EBI3/CCR7/CXCL9/CXCL13/CXCL11/CXCL10
regulation of T-helper 1 type immune response	-1.58389	0.044092	JAK3/XCL1/TNFSF4/IL1R1/SLAMF1/IL12B/CD80/IL27/CCL19
regulation of leukocyte apoptotic process	-1.58532	0.016978	JAK3/BCL3/RAG1/IL7R/CD274/IRF7/CD74/CCL5/IL10/CXCL12/IDO1/CCR7/CCL19
positive regulation of leukocyte chemotaxis	-1.59084	0.02226	IL4/SLAMF1/CD74/CCR6/CCL5/CXCL12/CCR7/CCL19/CXCL13/CXCL10
regulation of lymphocyte differentiation	-1.59115	0.00487	JAK3/RAG1/PTPN2/IL7R/CD86/TNFSF4/IL4/IFNB1/IL15/IL7/CD74/PRDM1/HLA-DRA/HLA-DRB1/IL12B/IFNG/SOCS1/IRF1/CD83/IL10/CD80/IL2RA/IRF4/IL27/LAG3/CCL19
lymphocyte differentiation	-1.59116	0.001484	JAK3/KLRC1/BCL3/RAG1/STAT3/PTPN2/IL7R/CD86/CTNNB1/PTGER4/TNFSF4/IL4/SLAMF1/IFNB1/IL15/IL7/CD74/PRDM1/HLA-DRA/HLA-DRB1/CCR6/ IL12B/RELB/IFNG/VCAM1/SOCS1/TNFSF13B/TNFRSF9/PAX5/IRF1/NFIL3/CD83/GPR183/IL10/CD80/IL2RA/IRF4/CCR7/IL27/LAG3/CCL19
antigen processing and presentation of exogenous antigen	-1.59663	0.026042	HLA-DQB1/TRAF6/TAP1/CD74/HLA-DPB1/HLA-DQA1/HLA-DRA/HLA-DPA1/HLA-DRB1/HLA-DOB/CD1A
negative regulation of leukocyte apoptotic process	-1.59665	0.026042	JAK3/BCL3/RAG1/IL7R/IRF7/CD74/CCL5/CXCL12/IDO1/CCR7/CCL19
regulation of T cell activation	-1.59721	0.001481	CD160/JAK3/DPP4/RAG1/JAK2/PTPN2/HLA-DQB1/IL7R/CD86/CTNNB1/XCL1/TNFSF4/

			TRAF6/IL4/SLAMF1/PDCD1LG2/IFNB1/CD274/IL15/IL7/CD74/HLA-DPB1/PRDM1/HLA-DQA1/HLA-DRA/HLA-DPA1/HLA-DRB1/HLA-DOB/IL12B/IFNG/ARG1/VCAM1/SOCS1/TNFSF13B/TNFRSF9/IRF1/CCL5/CD83/IL10/CASP3/CD70/CD80/IL2RA/IDO1/EBI3/IRF4/CCR7/IL27/LAG3/CCL19
positive regulation of T cell mediated cytotoxicity	-1.59747	0.048913	B2M/IL12RB1/XCL1/HLA-DRA/HLA-DRB1/IL12B/CD1A
regulation of cation transmembrane transport	-1.60562	0.02109	PML/KCNJ2/IFNG/ARG1/CXCL9/CXCL11/CXCL10
ion transmembrane transport	-1.61627	0.014634	PML/KCNJ2/IFNG/ARG1/CCR7/CXCL9/CCL19/CXCL11/CXCL10
cellular divalent inorganic cation homeostasis	-1.61899	0.019802	CCL5/CXCL12/CCR7/CXCL9/CCL19/CXCL11/CXCL10
alpha-beta T cell differentiation	-1.61995	0.004862	JAK3/BCL3/STAT3/CD86/PTGER4/TNFSF4/IL4/PRDM1/HLA-DRA/HLA-DRB1/IL12B/RELB/IFNG/SOCS1/IRF1/CD83/GPR183/CD80/IRF4/IL27/CCL19
regulation of alpha-beta T cell differentiation	-1.62382	0.019802	CD86/TNFSF4/IL4/PRDM1/HLA-DRA/HLA-DRB1/IL12B/IFNG/SOCS1/CD83/CD80/IRF4/IL27/CCL19
divalent inorganic cation homeostasis	-1.62831	0.014706	CCL5/CXCL12/CCR7/CXCL9/CCL19/CXCL11/CXCL10
T-helper 1 cell differentiation	-1.64343	0.022099	JAK3/TNFSF4/RELB/CD80/IL27/CCL19
regulation of receptor signaling pathway via JAK-STAT	-1.6475	0.015817	IFNA2/IL26/JAK3/BCL3/JAK2/PTPN2/IL7R/IL5/IL4/IFNB1/SOCS3/IL12B/IFNG/SOCS1/CCL5/IL10
regulation of T cell differentiation	-1.65196	0.003247	JAK3/RAG1/PTPN2/IL7R/CD86/TNFSF4/IL4/IFNB1/IL15/IL7/CD74/PRDM1/HLA-DRA/HLA-DRB1/IL12B/IFNG/SOCS1/IRF1/CD83/CD80/IL2RA/IRF4/IL27/LAG3/CCL19
positive regulation of CD4-positive, alpha-beta T cell activation	-1.65412	0.019031	HLA-DRB3/CD160/CD86/XCL1/TNFSF4/HLA-DRA/HLA-DRB1/IL12B/IFNG/SOCS1/CD83/CD80/CCL19
dendritic cell antigen	-1.66132	0.024119	HLA-DRB3/NOD1/CD74/HLA-DRA/HLA-DRB1/CCR7/CCL19

processing and presentation			
regulation of T-helper cell differentiation	-1.66503	0.017422	CD86/TNFSF4/IL4/HLA-DRA/HLA-DRB1/IL12B/CD80/IRF4/IL27/CCL19
positive regulation of ion transmembrane transport	-1.66764	0.024691	KCNJ2/IFNG/CXCL9/CXCL11/CXCL10
antimicrobial humoral response	-1.67527	0.02087	CXCL9/CXCL13/CXCL11/CXCL10
lymphocyte migration	-1.68359	0.010033	S1PR1/CCL23/CCL18/CCR6/CCL5/GPR183/CXCL12/CCR7/CCL19/CXCL13/CXCL11/CXCL10
cation transmembrane transport	-1.6927	0.005034	PML/KCNJ2/IFNG/ARG1/CCR7/CXCL9/CCL19/CXCL11/CXCL10
dendritic cell chemotaxis	-1.69278	0.020036	CXCR1/SLAMF1/CCR6/CCL5/GPR183/CCR7/CCL19
dendritic cell migration	-1.69278	0.020036	CXCR1/SLAMF1/CCR6/CCL5/GPR183/CCR7/CCL19
defense response to symbiont	-1.70027	0.001613	CD40/PRF1/MX1/IL4/IFNB1/IL15/IRF7/RELA/CASP1/PML/IL12B/IFIH1/TLR3/IFNG/IRF1/GBP1/CXCL9/IFITM1/IL27/IFIT2/CXCL10
T-helper 1 type immune response	-1.70568	0.006849	JAK3/BCL3/XCL1/TNFSF4/IL1R1/TRAF6/SLAMF1/HLA-DRB1/IL12B/RELB/CD80/EBI3/IL27/CCL19
positive regulation of lymphocyte migration	-1.7107	0.018116	CCL5/CXCL12/CXCL13/CXCL10
leukocyte apoptotic process	-1.71503	0.005034	JAK3/BCL3/RAG1/IL7R/CD274/IRF7/CD74/FAS/CCL5/IL10/CASP3/IL2RA/CXCL12/IDO1/CCR7/CCL19
response to chemokine	-1.75288	0.005017	CCL18/CCR6/CCL5/CXCL12/CCR7/CXCL9/CCL19/CXCL13/CXCL11/CXCL10
cellular response to chemokine	-1.75288	0.005017	CCL18/CCR6/CCL5/CXCL12/CCR7/CXCL9/CCL19/CXCL13/CXCL11/CXCL10
T-helper cell differentiation	-1.75424	0.004942	IL18/TBX21/RORC/IL21/IL12RB1/BATF/JAK3/BCL3/STAT3/CD86/PTGER4/TNFSF4/IL4/HLA-DRA/HLA-DRB1/IL12B/RELB/GPR183/CD80/IRF4/IL27/CCL19
T cell migration	-1.77538	0.005226	CCR6/CCL5/GPR183/CXCL12/CXCL13/CXCL11/CXCL10
regulation of CD4-positive,	-1.77766	0.006547	BATF/HLA-DRB3/CD160/JAK3/CD86/XCL1/TNFSF4/IL4/CD274/HLA-DRA/HLA-

alpha-beta T cell activation			DRB1/IL12B/IFNG/SOCS1/CD83/CD80/IL2RA/IRF4/IL27/CCL19
alpha-beta T cell activation involved in immune response	-1.78039	0.004878	JAK3/BCL3/STAT3/CD86/PTGER4/TNFSF4/IL4/HLA-DRA/HLA-DRB1/IL12B/RELB/IFNG/GPR183/CD80/IRF4/IL27/CCL19
CD4-positive, alpha-beta T cell differentiation	-1.85181	0.001672	JAK3/BCL3/STAT3/CD86/PTGER4/TNFSF4/IL4/HLA-DRA/HLA-DRB1/IL12B/RELB/IFNG/SOCS1/CD83/GPR183/CD80/IRF4/IL27/CCL19
regulation of T cell migration	-1.87098	0.005455	CCR6/CCL5/CXCL12/CXCL13/CXCL10
positive regulation of CD4-positive, alpha-beta T cell differentiation	-1.87882	0.003527	CD86/TNFSF4/HLA-DRA/HLA-DRB1/IL12B/IFNG/SOCS1/CD83/CD80/CCL19

Supporting information TABLE 6. List of monoclonal antibodies and other reagents

Antibody/Reagent	Source	Fluorochrome	Clone
Anti-human CD1a	BD Biosciences	PE	NA1/34
Anti-human CD14	Agilent Technologies	FITC	TÜK4
Anti-human HLA-DR	Biolegend	APC-fire	L243
Anti-human CD86	BD Biosciences	BV421	BU63
Anti-human PD-L1	BD Biosciences	BV421	MIH1
Anti-human CD209	BD Biosciences	APC	DCN46
Anti-human CD276	BD Biosciences	RB705	7-517
Anti-human TGFβ/LAP	BD Biosciences	BV421	TW4-9E7
Anti-human CD3	BD Biosciences	BV510	SP34-2
Anti-human CD4	BD Biosciences	APC	SK3
Anti-human CD8	BD Biosciences	BV421	RPA-T8
Anti-human CD69	BD Biosciences	RB705	FN50
Anti-human CD25	BD Biosciences	RB705	2A3
Anti-human FoxP3	BD Biosciences	PE	236A/E7
Anti-human CTLA4	BD Biosciences	PE	BNI3
Anti-human LAG3	BD Biosciences	BV421	T47-530

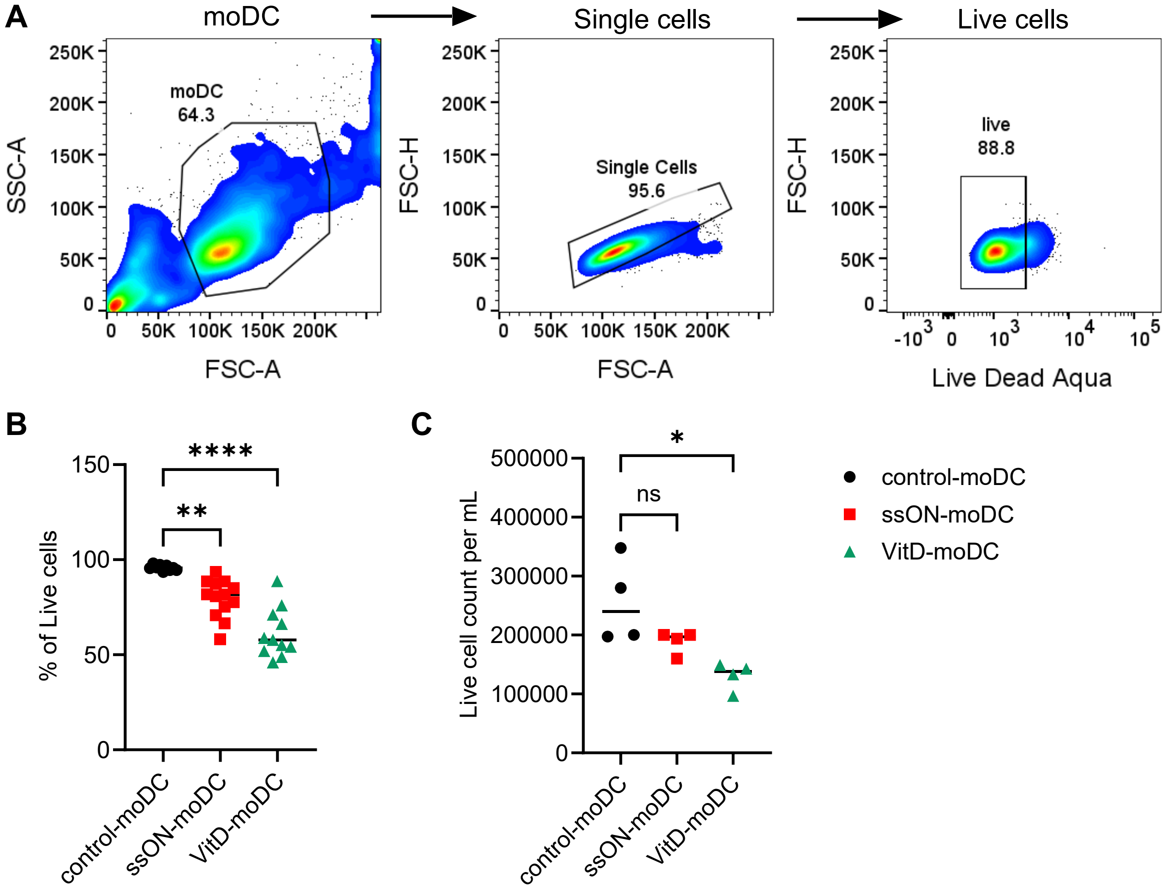

FIGURE S1. The percentage of live moDCs on day 6 after treatment with ssON or VitD compared to control-moDCs. (A) Gating Strategy to gate on moDCs where single cells were gated and dead cells were excluded by using a live-dead stain Aqua. The dead cells were identified as Aqua positive and excluded from analysis related to Figure 1. (B) Frequency of live control-, ssON-, and VitD-moDCs using the gating strategy shown above. (C) Viable cell count per mL was determined using trypan blue and counting non-blue cells using a hemocytometer under the light microscope. Data representative of >3 independent experiments, including n>4 individuals. Multiple comparisons were made using one-way ANOVA (Kruskal-Wallis test). ****p<0.0001, **p<0.01.*p<0.05, ns>0.05.

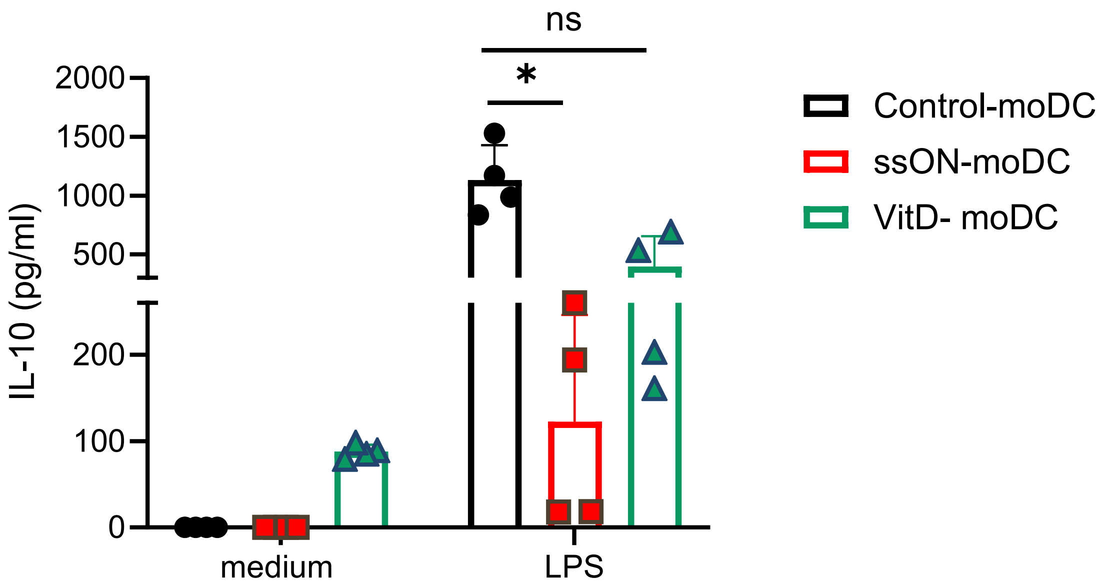

FIGURE S2. IL-10 was not released in moDCs differentiated in the presence of ssON. Cell culture supernatants from LPS-stimulated moDCs (24 hours) were analyzed using ELISA to measure the levels of IL-10. Data are representative of two independent experiments, including n=4 individuals. IL-10 production increased upon LPS-stimulation of moDCs. Multiple comparisons were made using one-way ANOVA (Kruskal-Wallis test). All data are biological replicates and are shown as mean ± SEM, n=4 donors *p<0.05, ns>0.05.

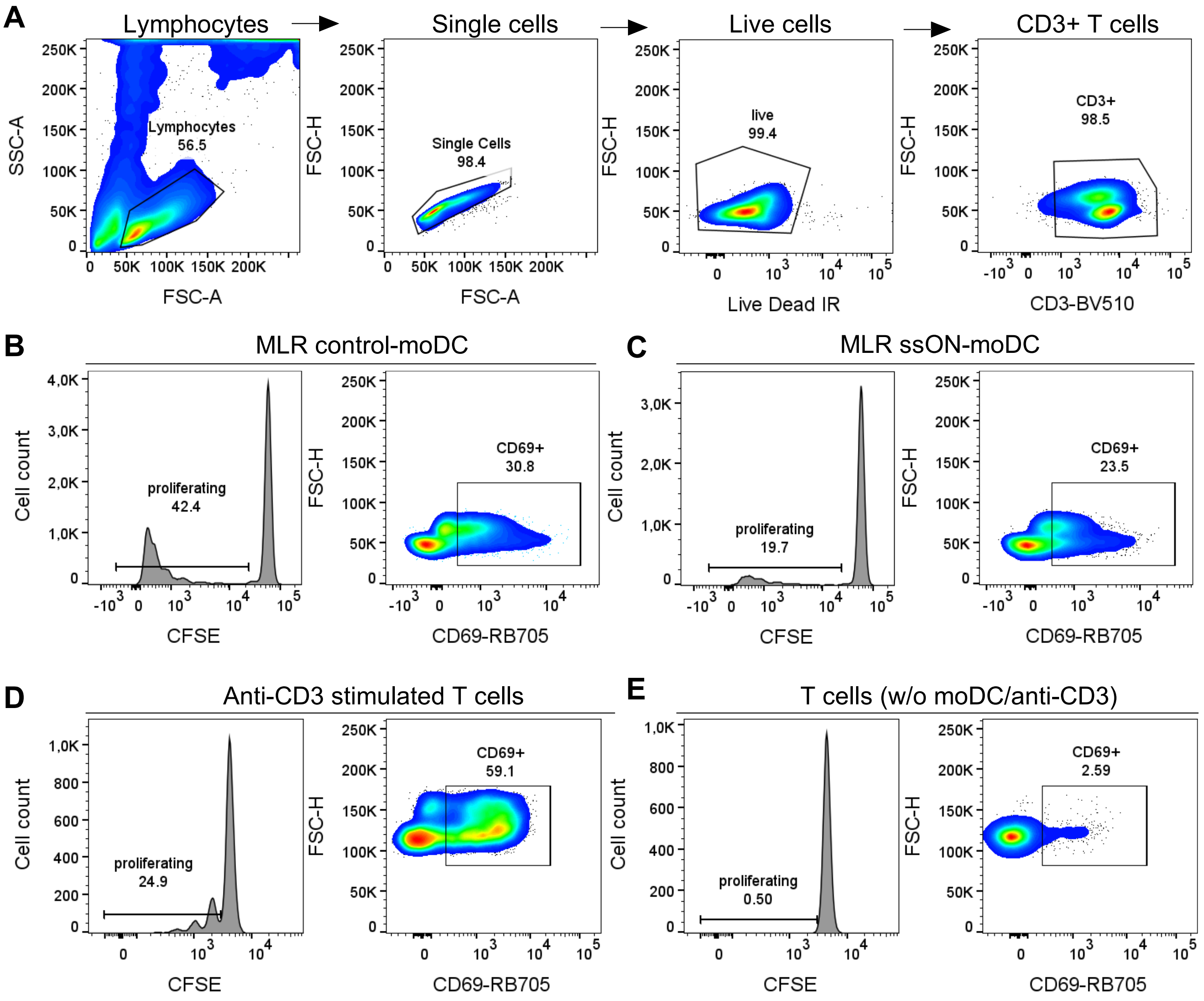

FIGURE S3. Gating strategy to measure CD69 expression in allogenic T cells. On day 6, control- or ssON-moDCs were co-cultured with CFSE-labelled allogeneic T cells at 1:2 ratio in 96-well round-bottom plates and incubated for an additional 6 days. (A) MLR co-cultures were analyzed on day 12 using flow cytometry. The initial gate was set on lymphocytes, followed by gating on single cells. Then, dead cells were excluded using a live-dead dye, and the final gate was set to identify $CD3^{+}$ T cells. (B-E) The histogram plots show proliferating $CD3^{+}$ T cells identified as $CFSE^{low}$ T cells. Furthermore, CD69 expression was evaluated in $CD3^{+}$ T cells cultured with (B) control- or (C) ssON-moDCs on day 12. To further confirm allogeneic T cell activation by moDCs, we compared CD69 expression in T cells activated with an antibody targeting the CD3 complex (positive control) (D) or unstimulated T cells (negative control) (E), cultured for three days.

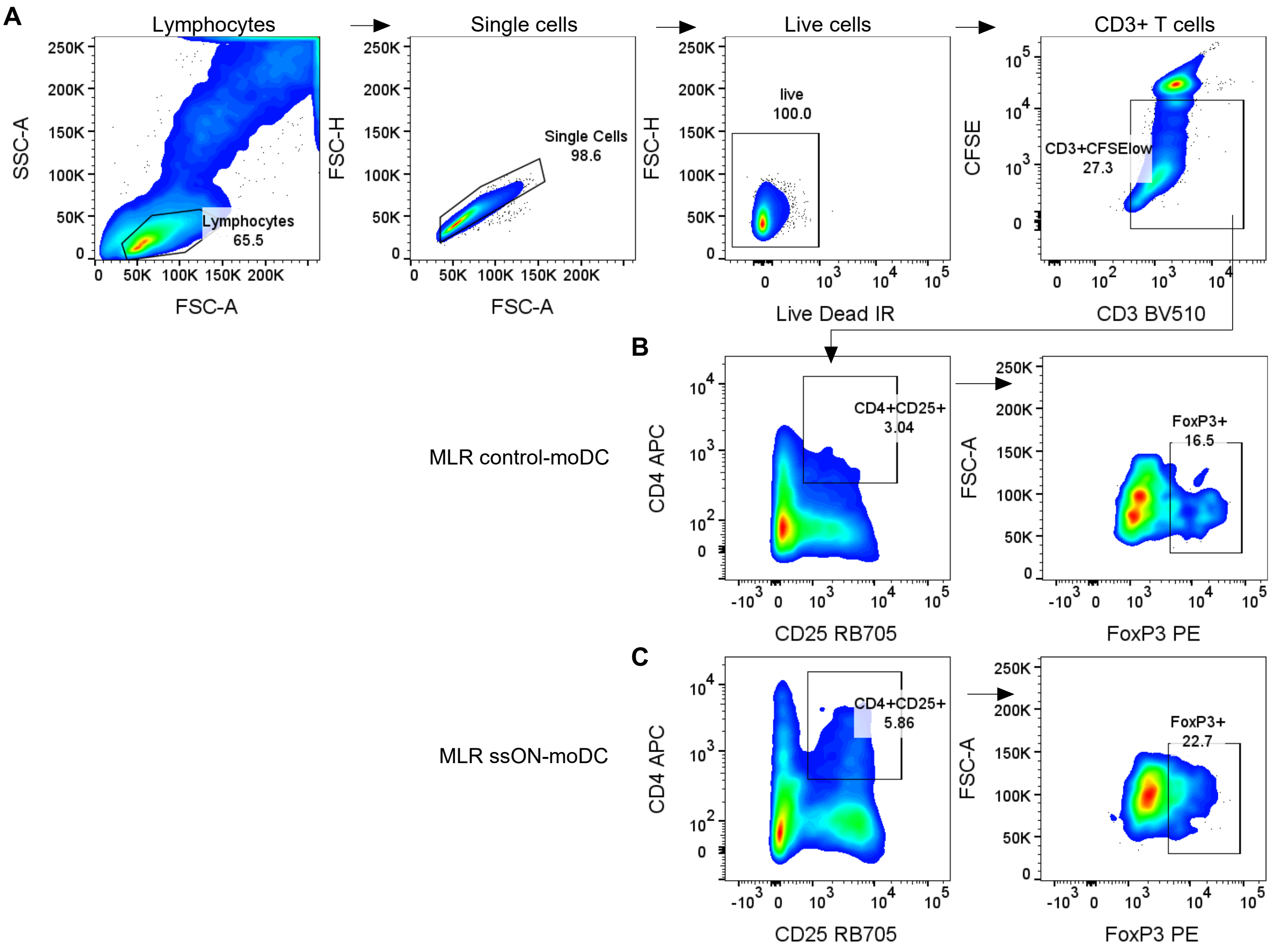

FIGURE S4. Gating strategy for measuring the frequency of $FoxP3^{+}$ Tregs in MLR cultures. (A) Initial gates were set on lymphocytes, followed by single and live cells. Proliferating T cells were identified as $CD3^{+}CFSE^{low}$ cells. Further $CD4^{+}CD25^{+}$ cells were gated amongst the $CD3^{+}CFSE^{low}$ cells. The frequency of $FoxP3^{+}$ Tregs was determined in MLR co-cultures containing (B) control-moDCs and (C) ssON-moDCs amongst the proliferating T cells.

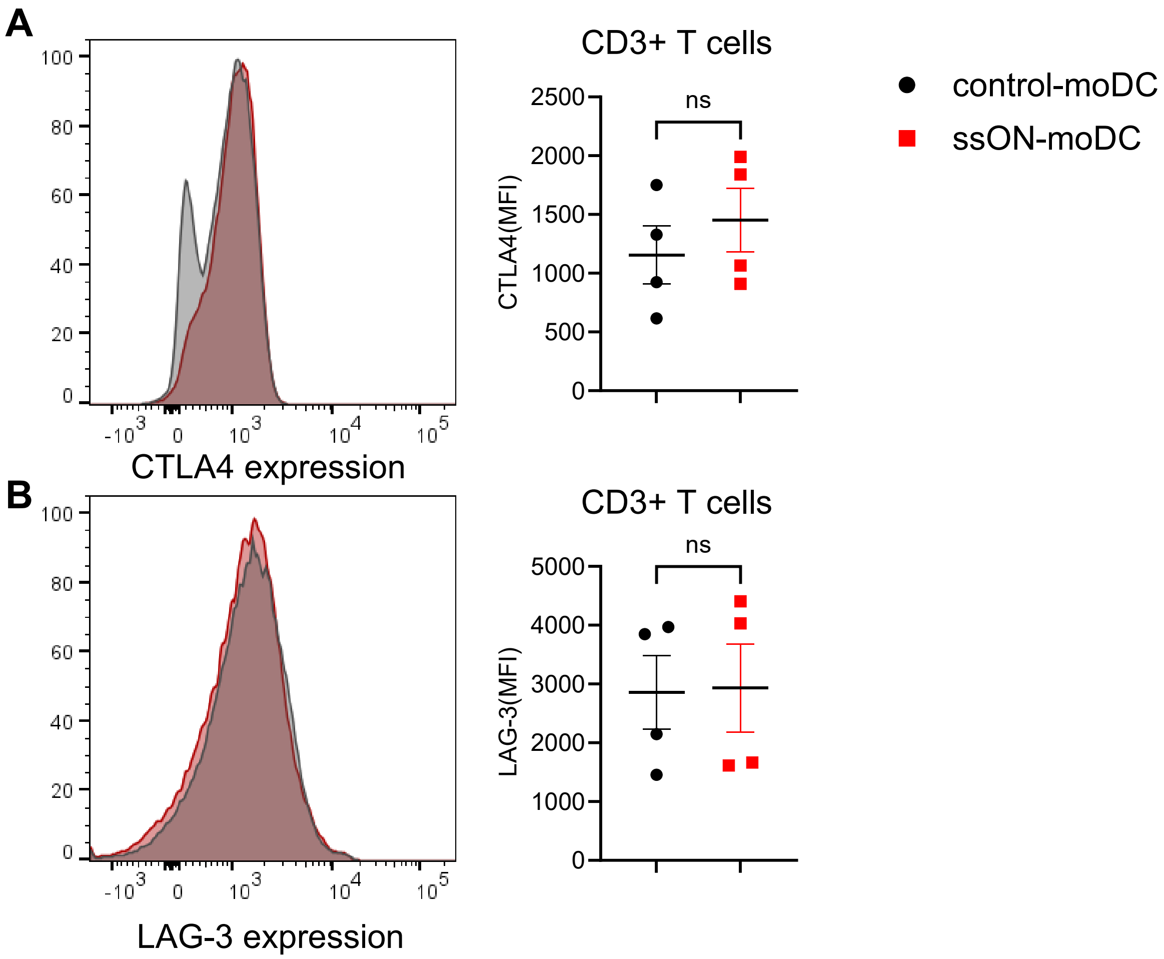

FIGURE S5. CTLA4 and LAG-3 expression remain unchanged in allogenic T cells cultured in the presence of ssON-moDCs. $CD3^{+}$ T cells were gated as shown previously in Figure S3A. Histogram plots depicting (A) CTLA4 and (B) LAG-3 expression in T cells cultured with control- (black) or ssON-moDCs (red). Bar graphs showing the MFI values where data were obtained from three independent experiments. Pairwise comparisons were made using the Mann-Whitney test. All data are biological replicates and are shown as mean $\pm$SEM, n=5-6 donors *p<0.05.

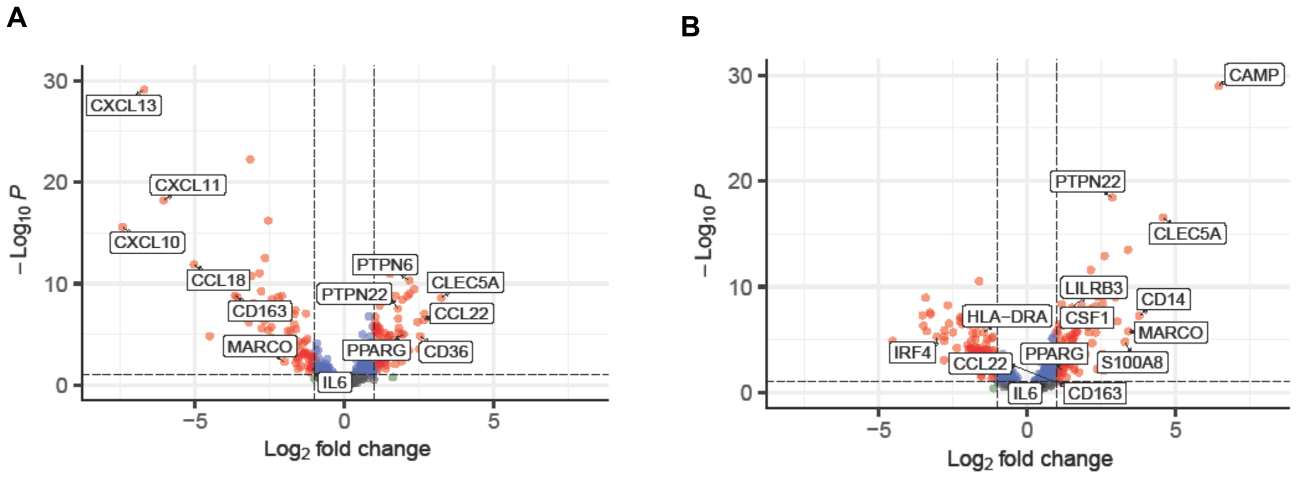

FIGURE S6: Differentially expressed genes in ssON-moDCs and VitD-moDCs after stimulation with LPS for 24 hours. The NanoString technique was used to assess changes in the transcriptome. Volcano plots show DEGs where the vertical lines show the threshold of Log2foldchange>0.5 and horizontal lines show threshold *p*<0.01 in (A) ssON-moDCs (B) VitD-moDCs as compared with LPS-stimulated control-moDCs. Data are representative of two experiments with n=4 individuals.

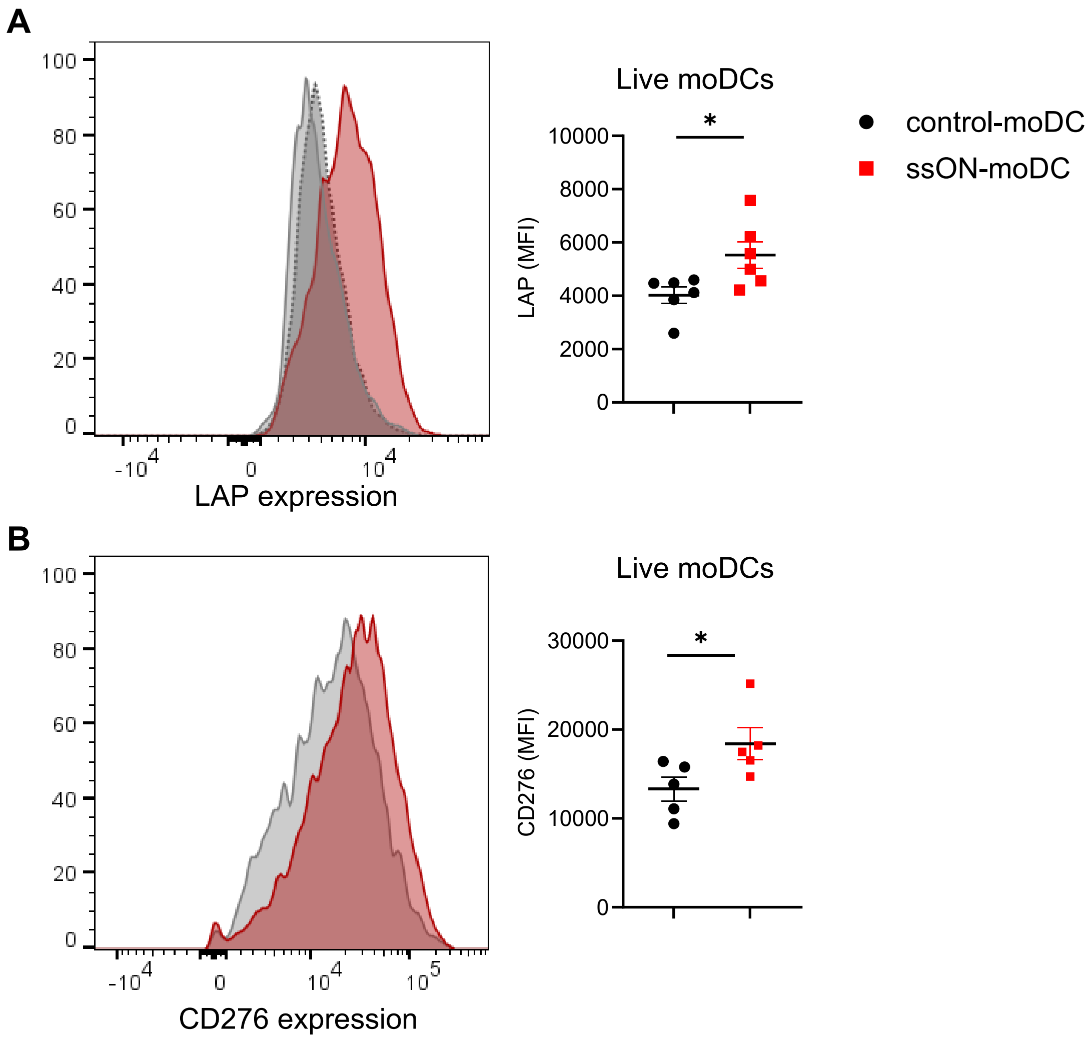

FIGURE S7. Expression of additional tolerogenic markers in ssON-moDCs. The gating strategy was used as shown before in Figure S1A. On day 6, expression of (A) LAP (grey dotted line represents isotype) (B) CD276 was significantly increased in ssON-moDCs (red peak) as compared to control-moDCs (grey peak). Pairwise comparisons were made using the Mann-Whitney test. All data are biological replicates from three independent experiments and are shown as mean ± SEM, n=5-6 donors $*p<0.05$.

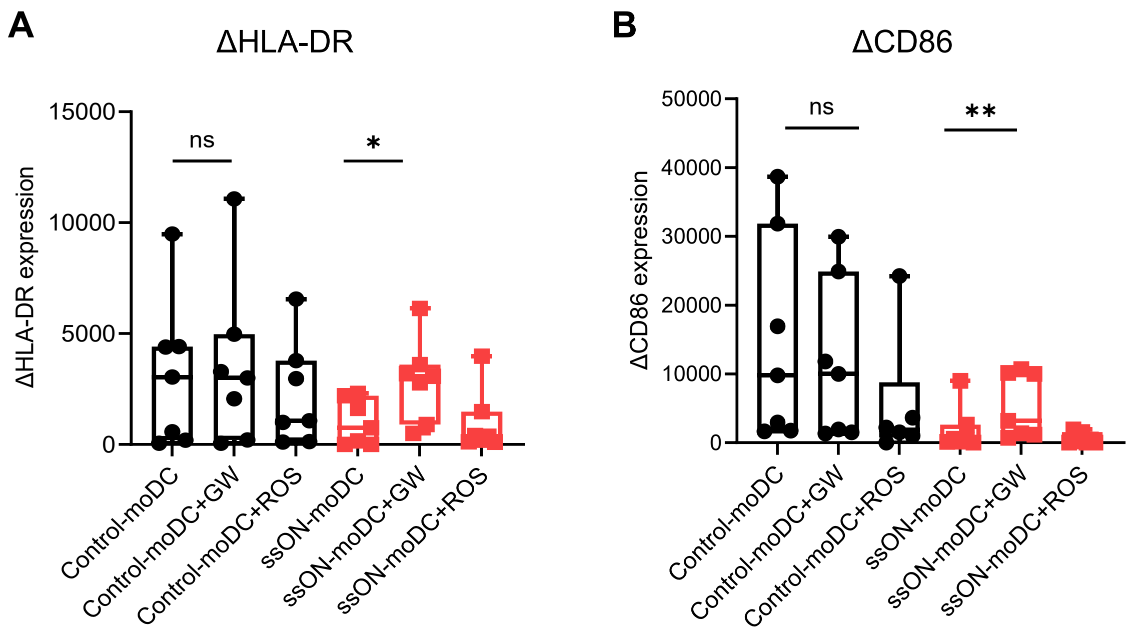

FIGURE S8: Changes in LPS-responsiveness using either the inhibitor GW9662 (GW) or activator rosiglitazone (ROS) of PPARγ. (A) MFI quantification showing the changes in HLA-DR expression 24 hours after LPS-stimulation in the presence of PPARγ antagonist GW or agonist ROS in either control-moDC (black) or ssON moDC (red). (B) MFI quantifications showing the changes in expression of CD86 on day 7, 24 hours after LPS-stimulation in the presence of GW or ROS in either control-moDCs (black) or ssON-moDCs (red). Data were obtained and pooled from n=5-6 donors from three independent experiments. Pairwise comparisons were made using the Mann-Whitney test. All data are biological replicates pooled from three independent experiments and are shown as mean $\pm$ SEM, ns $p>0.05$ *$p<0.05$, **$p<0.01$.
